# USP27X negatively regulates antiviral signaling by deubiquitinating RIG-I

**DOI:** 10.1371/journal.ppat.1008293

**Published:** 2020-02-06

**Authors:** Xinyue Tao, Bei Chu, Di Xin, Lin Li, Qinmiao Sun

**Affiliations:** 1 State Key Laboratory of Membrane Biology, Institute of Zoology, Chinese Academy of Sciences, Beijing, China; 2 Savaid Medical School, University of Chinese Academy of Sciences, Beijing, China; 3 School of Life Sciences, University of Science and Technology of China, Hefei, Anhui, China; University of Pennsylvania Perelman School of Medicine, UNITED STATES

## Abstract

RIG-I plays important roles in pathogen sensing and activation of antiviral innate immune responses in response to RNA viruses. RIG-I-mediated signaling must be precisely controlled to maintain innate immune signaling homeostasis. Previous studies demonstrated that lysine 63 (K63)-linked polyubiquitination of RIG-I is vital for its activation, but the mechanisms through which RIG-I is deubiquitinated to control innate immune responses are not well understood. Here we identified USP27X as a negative regulator of antiviral signaling in response to RNA viruses through siRNA library screening. Further functional studies indicated that USP27X negatively modulated RIG-I-mediated antiviral signaling in a deubiquitinase-dependent manner. Mechanistically, we found that USP27X removed K63-linked polyubiquitin chains from RIG-I to negatively modulate type I interferon signaling. Collectively, these studies uncover a novel negative regulatory role of USP27X in targeting RIG-I to balance innate immune responses.

## Author summary

Innate antiviral immunity provides the first line of the host defense system that rapidly detects invading virus through pattern recognition receptors, and subsequently inducing type I interferon production. Protein ubiquitination/deubiquitination play important roles in antiviral signaling, however how the dynamic regulation of ubiquitin modification controls innate immune signaling against RNA virus infection remains unclear. In this study, we screened E3 ligases and deubiquitinases (DUBs) using a siRNA library, and identified USP27X, a DUB, as a negative regulator of antiviral signaling in response to RNA virus infection. Moreover, we employed knockdown and knockout of USP27X various cell lines to perform functional analyses, and found that USP27X suppressed RIG-I-mediated antiviral signaling in a DUB activity-dependent manner. Mechanistically, we found that USP27X removed K63-linked polyubiquitin chains from RIG-I to negatively modulate type I IFN signaling. Collectively, our study reveals that in addition to removing K48-linked polyubiquitin chains, USP27X can target RIG-I for removing the K63-linked polyubiquitin chains, thereby balancing antiviral signaling to avoid deleterious effects on host cells.

## Introduction

Viral infection can trigger innate immune responses, which serve as the first line of host defense in detecting and eliminating viruses. Innate immune responses are triggered by various pattern recognition receptors sensing different pathogen-associated molecular patterns [1, 2]. Host cells use different receptors to recognize RNA and DNA viruses. Toll-like receptors (TLRs)-3/7/8 and retinoic-acid-inducible gene I (RIG-I)-like receptors (RLRs), including RIG-I and melanoma differentiation-associated protein 5 (MDA5), serve as detectors of RNA viruses [3, 4]. TLR9 and cytoplasmic DNA sensors such as gamma-interferon-inducible protein 16 (IFI16), DNA-dependent activator of interferon-regulatory factor (DAI), DEAD-box helicase 41(DDX41), and cyclic GMP-AMP synthase (cGAS) play important roles in DNA virus recognition [5]. These receptors recruit various adaptor proteins such as MAVS (also known as IPS1, VISA or CARDIF), MYD88, TRIF or STING (also known as MITA or ERIS) to activate downstream signaling pathways and subsequently inducing type I interferon (IFN) production [4, 5].

RIG-I and MDA5 function as cytosolic viral RNA sensors. Their structures are very similar: both consist of two caspase activation and recruitment domains (CARDs), a central DexD/H helicase domain and a C-terminal regulatory domain (RD) [6, 7]. Viral infection can induce RIG-I conformational changes resulting in exposure of CARDs from an autorepressed state [6–9]. RIG-I is further activated by K63-linked ubiquitin chains, recruiting and activating MAVS through their CARDs [10, 11]. Subsequently, activated MAVS recruits TANK-binding kinase 1 (TBK1) and the IκB kinase (IKK) complex to activate the transcription factors interferon regulatory factor 3/7 (IRF3/7) and NF-κB, respectively, which coordinate to induce expression of IFNs and pro-inflammatory cytokines [12–15].

Protein ubiquitination plays a pivotal role in regulating RIG-I-mediated signaling. Previous studies found that K63-linked polyubiquitination of RIG-I was required for activation and downstream signaling transduction, while K48-linked polyubiquitination regulated RIG-I stability and inhibited type I IFN production [16]. TRIM25 was the first E3 ligase shown to be involved in K63-linked ubiquitination of RIG-I [10]. Further studies indicated that a number of other factors were also involved in K63-linked ubiquitination of RIG-I such as Riplet/RNF135, TRIM4 and MEX3C [17–19]. Additionally, RNF125, RNF122, CHIP and c-Cbl were identified as E3 ligases involved in K48-linked ubiquitination of RIG-I [20–23]. Moreover, several deubiquitinases (DUBs) including CYLD [24], USP21 [25] and USP3 [26] were found to remove K63-linked polyubiquitin chains from RIG-I, while USP4 [27] removed K48-linked polyubiquitin chains from RIG-I. The mechanisms through which RIG-I ubiquitination and deubiquitination are tightly balanced to maintain innate immune response homeostasis are still not fully understood.

USP27X is a DUB and a member of the cysteine protease family. A recent study suggested that USP27X, together with USP22 and USP51, regulate monoubiquitination of histone H2B [28]. Moreover, USP27X stabilizes Bim through deubiquitination, subsequently enhancing apoptosis [29]. USP27X was also found to play an important role in cell migration and chemoresistance through stabilization of Snail1 proteins [30]. Collectively, these studies indicated that USP27X plays primary roles in removal of K48-linked polyubiquitin chains, subsequently regulating protein stability. However, whether USP27X has other biological functions or whether it plays a role in removing K63-linked polyubiquitin chains remains largely unknown.

To better understand the roles and molecular mechanisms of ubiquitination in RIG-I-mediated signaling, we screened E3 ligases and DUBs using a siRNA library, and identified USP27X as a negative regulator of antiviral signaling. We employed knockdown and knockout of USP27X various cell lines, performed further functional analyses, and demonstrated that USP27X inhibited RIG-I-mediated antiviral signaling in a DUB activity-dependent manner. Moreover, we found that USP27X removed K63-linked RIG-I polyubiquitin chains to negatively regulate type I IFN signaling. Our results demonstrate that in addition to removing K48-linked polyubiquitin chains, USP27X can remove K63-linked polyubiquitin chains from RIG-I, thus negatively modulating antiviral signaling to maintain innate immune response homeostasis.

## Results

### Identification of USP27X as a negative regulator of type I IFN signaling

**Fig 1:**
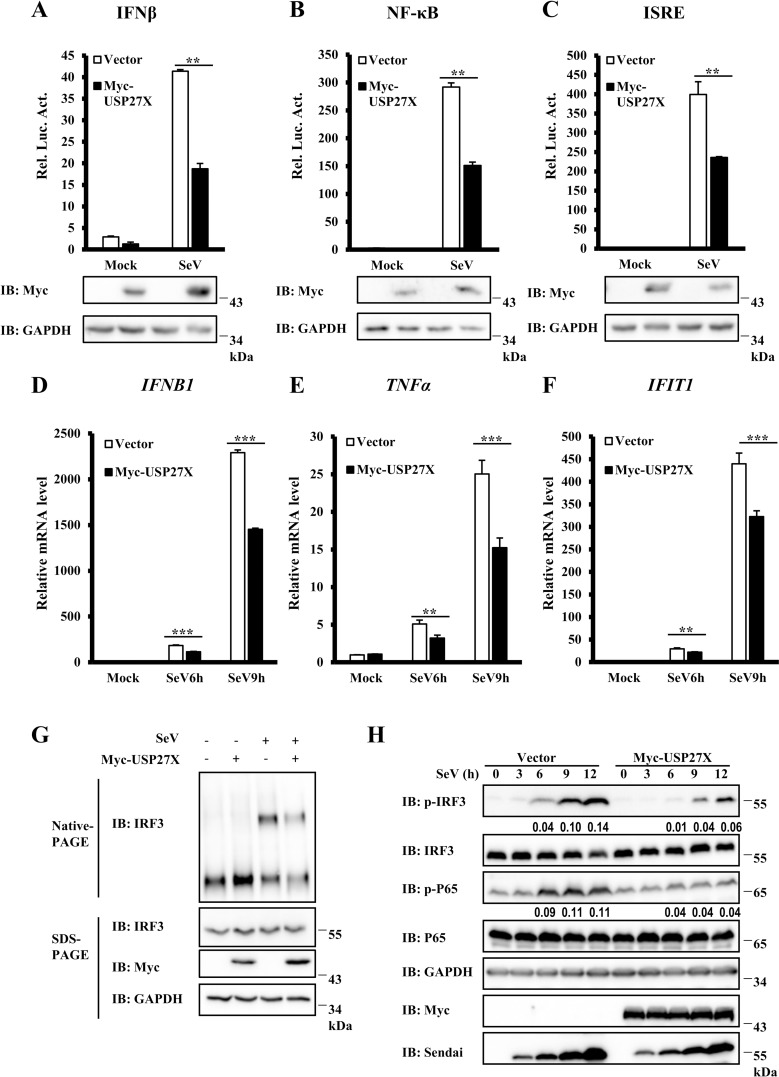
Identification of USP27X as a negative regulator of antiviral signaling. (A–C) HEK293T cells were co-transfected with the indicated expression plasmids and luciferase reporter constructs driven by promoters of IFNβ (A), NF-κB (B), or ISRE (C), as well as Renilla as an internal control. Twenty-four hours after transfection, cells were infected with SeV for 12 h. Cell lysates were analyzed for luciferase assays (upper panel) and immunoblotting assays (lower panels). (D–F) HEK293T cells were transfected with plasmids expressing USP27X or empty vector. Twenty-four hours after transfection, the cells were infected with SeV for the indicated times, and abundance of mRNAs encoding IFNB1 (D), TNFα (E) and IFIT1 (F) was measured by qRT-PCR. (G) HEK293T cells were transfected with plasmids expressing USP27X or empty vector. Twenty-four hours after transfection, the cells were infected with SeV for 9 h. Cell lysates were resolved by native gel electrophoresis (upper panel) or SDS-PAGE (lower panels) and analyzed with the indicated antibodies. (H) HEK293T cells were transfected with plasmids expressing USP27X or empty vector. Twenty-four hours after transfection, the cells were infected with SeV for the indicated times, followed by immunoblotting. The data shown in (A–F) are from one representative experiment of at least three independent experiments [mean ± SD of duplicate experiments in (A–C) or triplicate experiments in (D–F)]. The two-tailed Student’s t-test was used to analyze statistical significance. ** P < 0.01; *** P < 0.001 versus control groups.

To better understand the role of ubiquitination in antiviral innate immune signaling, we performed a genome-wide siRNA screen using the Silencer Human Ubiquitin siRNA Library to identify novel ubiquitin-conjugating enzymes, E3 ligases or DUBs involved in regulating antiviral innate signaling. We used a human embryonic kidney 293T (HEK293T) stable reporter cell line expressing firefly luciferase driven by a human IFNβ promoter for screening. From this screening, we identified a DUB, USP27X, which acted as a negative regulator of IFNβ expression induced by Sendai virus (SeV) infection (S1 Fig). To further examine the function of USP27X in antiviral signaling, we overexpressed USP27X together with an IFNβ luciferase reporter. Following SeV infection, we found that overexpression of USP27X significantly inhibited SeV-induced IFNβ activation (Fig 1A). Since IFNβ activation requires cooperation of both the IRF3 and NF-κB pathways, we employed an IFN-stimulated response element (ISRE) luciferase reporter, which is sufficiently activated by IRF3 activation, or a NF-κB luciferase reporter, to examine how USP27X modulates IFNβ signal transduction. As shown in Fig 1B and 1C, overexpression of USP27X significantly reduced the activation of NF-κB and IRF3 promoters induced by SeV infection. Consistently, quantitative reverse-transcription polymerase chain reaction (qRT-PCR) assays indicated that ectopic USP27X expression significantly decreased transcriptional levels of antiviral genes such as *IFNB1*, *TNFα* and *IFIT1* following SeV infection (Fig 1D–1F). Since dimerization and phosphorylation of IRF3 are required for activation of type I IFN signaling, we next examined whether ectopic expression of USP27X affected dimerization and phosphorylation of IRF3 induced by SeV infection. As shown in Fig 1G and 1H, USP27X overexpression significantly decreased IRF3 dimerization and phosphorylation in response to SeV infection. In addition, we observed that phosphorylation of P65 induced by SeV infection was also significantly inhibited by USP27X overexpression (Fig 1H). Moreover, overexpression of USP27X evidently inhibited nuclear translocation of IRF3 and P65 upon SeV infection (S2 Fig). Taken together, these data suggested that USP27X potentially functioned as a negative regulator of antiviral signaling.

Previous studies have shown that USP27X could employ another start site (CTG) upstream of the predicted ATG start site to code a long form (72-kDa) of USP27X (hereafter referred as USP27X-72) (S3A Fig). Compared with the 49-kDa USP27X, the USP27X-72 contains additional N-terminal 198 amino acids [28][30]. We next examined whether USP27X-72 functionally affects the RIG-I-mediated antiviral signaling. As shown in S3B–S3D Fig, similar to USP27X, expression of USP27X-72 in HEK293T cells significantly reduced activation of IFNβ, ISRE and NF-κB following SeV infection, suggesting that the additional 198 amino acids at the N-terminus is not essential for the function of USP27X. In agreement with this, we found that overexpression of the 198 amino acids fragment (USP27X-72 (N)) failed to inhibit the activation of IFNβ, ISRE and NF-κB following SeV infection (S3B–S3D Fig).

### USP27X knockdown increases antiviral signaling

**Fig 2:**
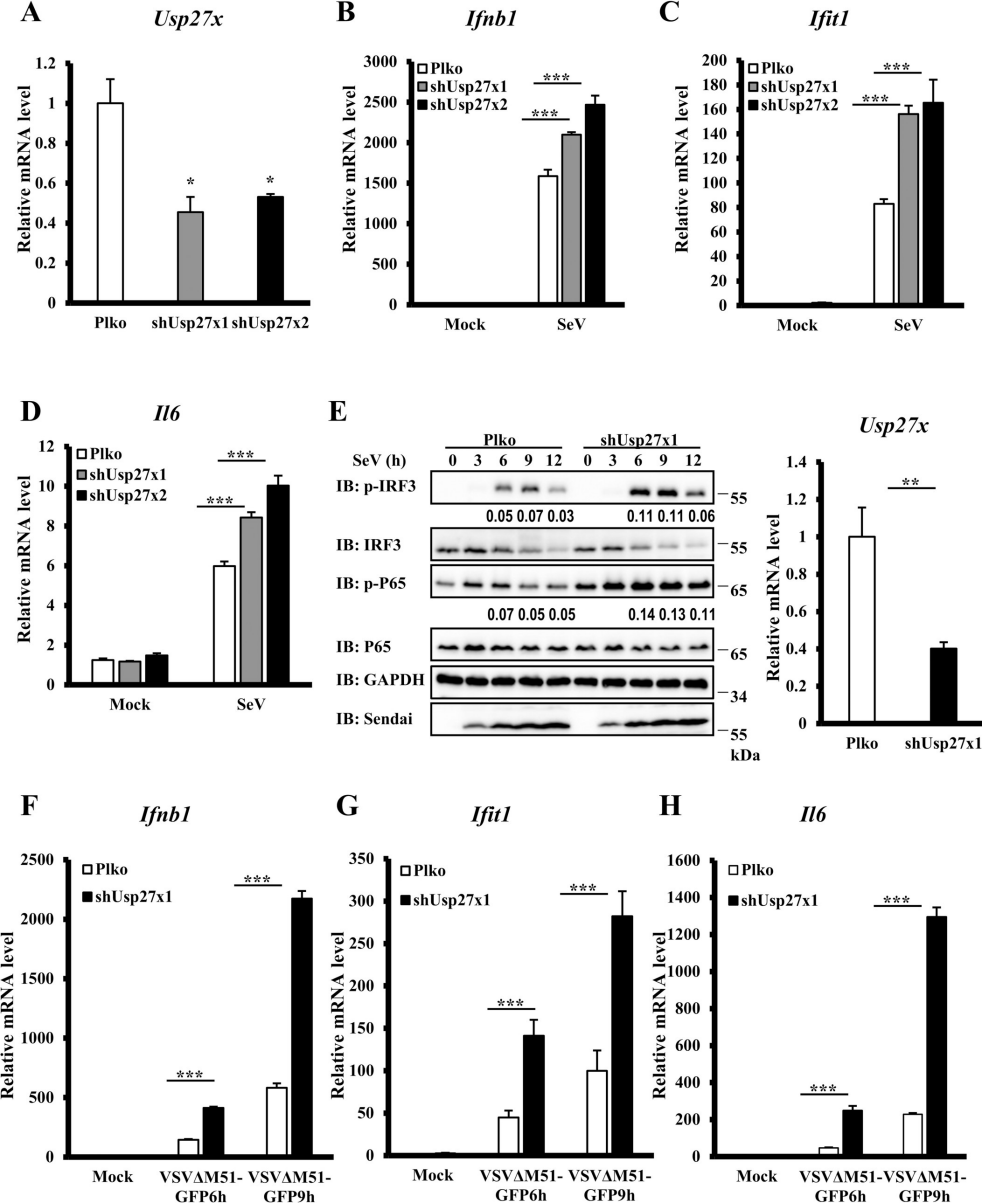
Knockdown of USP27X enhances type I IFN signaling. (A–D) RAW264.7 cells were infected with lentiviral vectors targeting two different regions of mUsp27x (shUsp27x1, shUsp27x2) or empty vector for 48 h, followed by SeV infection for 9 h. The cells were collected for qRT-PCR assays to measure levels of *Usp27x* (A), *Ifnb1* (B), *Ifit1* (C) and *Il6* (D) mRNA. (E) RAW264.7 cells were infected with lentiviral vectors targeting Usp27x (shUsp27x1) or empty vector for 48 h, followed by SeV infection for the indicated times. The cells were collected for qRT-PCR assays to measure levels of Usp27x mRNA (right panel) or lysed for immunoblotting with the indicated antibodies (left panels). (F–H) RAW264.7 cells were infected with lentiviral vectors targeting Usp27x (shUsp27x1) or empty vector for 48 h, followed by VSVΔM5-GFP infection at an MOI of 1 for the indicated times. The cells were collected for qRT-PCR assays to measure levels of *Ifnb1* (F), *Ifit1* (G) and *Il6* (H) mRNA. The data shown in (A–H) are from one representative experiment of at least three independent experiments (mean ± SD of triplicate experiments). The two-tailed Student’s t-test was used to analyze statistical significance. * P < 0.05; **P < 0.01; *** P < 0.001 versus control groups.

To further examine the biological functions of endogenous USP27X in modulating antiviral signaling, we employed two lentiviral-delivered shRNAs specifically targeting non-overlapping regions of the coding region of Usp27x, and tested whether knockdown of Usp27x affected antiviral signaling in mouse macrophage RAW 264.7 cells. As shown in Fig 2A–2D, both shRNAs efficiently reduced Usp27x mRNA levels, and Usp27x knockdown significantly enhanced expression of *Ifnb1*, *Ifit1 and Il6* mRNA following SeV infection compared with control cells. Consistent with the qRT-PCR results, levels of phosphorylated IRF3 and P65 were increased in Usp27x-knockdown cells following SeV infection (Fig 2E). To further study the function of USP27X, we used another RNA virus, vesicular stomatitis mutant virus (VSVΔM51-GFP) carrying a single amino acid deletion (Met51) in the matrix protein in VSV-GFP virus, and again observed increased abundance of *Ifnb1*, *Ifit1 and Il6* mRNA in Usp27x knockdown cells following VSVΔM51-GFP infection compared with control cells (Fig 2F–2H). To further determine whether USP27X specifically affects antiviral signaling, we examined the role of USP27X in modulating the TLR3/4-mediated signaling in RAW 264.7 cells. As shown in S4 Fig, we found that knockdown of UPS27X failed to enhance phosphorylation of IRF3 and P65 upon treatment of Poly(I:C) (TLR3 ligand) or LPS (TLR4 ligand). In addition, we examined expression levels of USP27X in multiple human cell lines including HEK293T, HeLa, THP-1, HepG2, HCT116 and H1299 cells (S5A Fig) and found that *USP27X* transcripts displayed the highest levels in HepG2 cells when compared to other tested cell lines. Functional analysis revealed that enhanced levels of *IFNB1*, *TNFα* and *IFIT1*mRNA were observed in USP27X knockdown HepG2 cells upon SeV infection compared with control cells (S5B–S5E Fig). Taken together, these findings support a notion that USP27X negatively modulated antiviral responses.

### USP27X knockout enhances antiviral signaling

**Fig 3:**
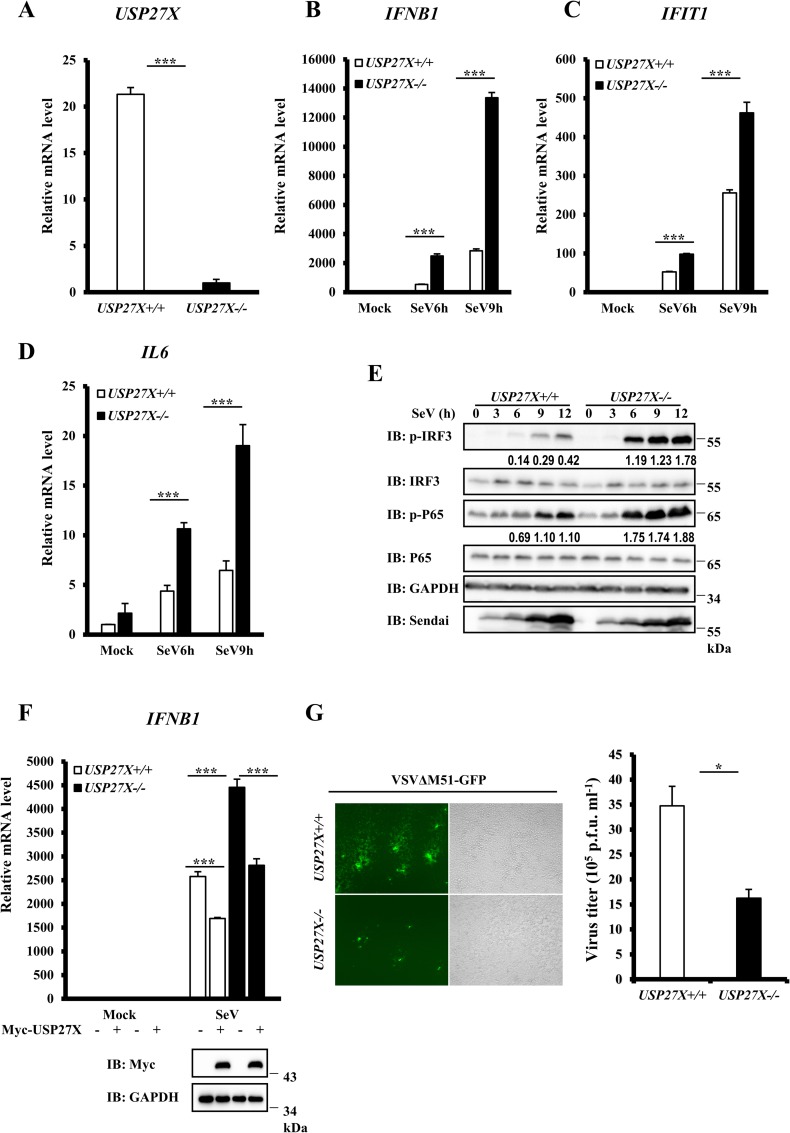
Knockout of USP27X increases type I IFN signaling. (A–D) HEK293T *USP27X*^*+/+*^ and *USP27X*^*-/-*^ cells were infected with SeV for the indicated times, then lysed for measurement of *USP27X* (A), *IFNB1* (B), *IFIT1* (C) and *IL6* (D) mRNA levels by qRT-PCR. (E) HEK293T *USP27*X^+/+^ and *USP27*X^-/-^ cells were infected with SeV for the indicated times, then lysed for immunoblotting with the indicated antibodies. (F) HEK293T *USP27X*^+/+^ and *USP27*X^-/-^ cells were transfected with USP27X expression plasmid or empty vector. Twenty-four hours after transfection, the cells were infected with SeV for 9 h, followed by measurement of *IFNB1* mRNA levels by qRT-PCR. (G) HEK293T *USP27X* ^+/+^ and *USP27X*^-/-^ cells were infected with VSVΔM51-GFP at an MOI of 0.01 for 9 h. Culture supernatants were collected to measure viral titers by plaque assay. The data shown in (A–D) and (F–G) are from one representative experiment of at least three independent experiments [mean ± SD of triplicate experiments in (A–D, F) or duplicate experiments in (G)]. The two-tailed Student’s t-test was used to analyze statistical significance. * P < 0.05; *** P < 0.001 versus control groups.

To further understand the biological function of USP27X, we generated stable USP27X-knockout HEK293T cells and investigated whether USP27X knockout affected antiviral signaling. Since we failed to detect endogenous USP27X expression using commercial antibodies, USP27X knockout clones were verified by DNA sequencing, and knockout was confirmed by measurement of *USP27X* mRNA abundance using qRT-PCR (Fig 3A). As shown in Fig 3B–3D, qRT-PCR assays revealed markedly higher levels of *IFNB1*, *IFIT1* and *IL6* mRNA in USP27X knockout cells following SeV infection compared with control cells. Consistently, we observed that levels of phosphorylated IRF3 and P65 were elevated in USP27X knockout cells (Fig 3E). To examine the specific role of USP27X in antiviral signaling, we performed rescue experiments and found that overexpression of USP27X reversed the enhancement of *IFNB1* mRNA induced by SeV infection in *USP27X*^*-/-*^ cells (Fig 3F). Consistently, knockout of USP27X in human HeLa cells also enhanced the levels of *IFNB1*, *IFIT1* and *IL6* mRNA induced by SeV infection or PolyI:C transfection (S6A Fig). We also found that loss of USP27X in HepG2 cells enhanced the levels of *IFNB1*, *IFIT1* and *TNFα* mRNA induced by SeV infection (S6B Fig), and increased nuclear translocation of IRF3 and P65 (S7 Fig). In addition, we generated Usp27x-knockout mouse fibroblast L929 and macrophage RAW 264.7 cells and found that the abundance of *Ifnb1*, *Ifit1* and *Il6* mRNA in these cells was higher following SeV infection compared with control cells (S6C and S6D Fig). These data further indicated that USP27X negatively mediated antiviral type I IFN signaling.

Since type I IFN signaling plays an important role in viral amplification [31], we next determined whether USP27X was involved in regulating viral amplification. The titers of the vesicular stomatitis mutant virus (VSVΔM51-GFP) were significantly lower in USP27X-knockout HEK293T cells than in control cells (Fig 3G). Consistent results were obtained in USP27X-knockout HepG2 cells (S8 Fig). Taken together, these data provide further biological evidence that USP27X played a negative regulatory role in cellular antiviral responses.

### USP27X targets RIG-I to regulate antiviral signaling

**Fig 4:**
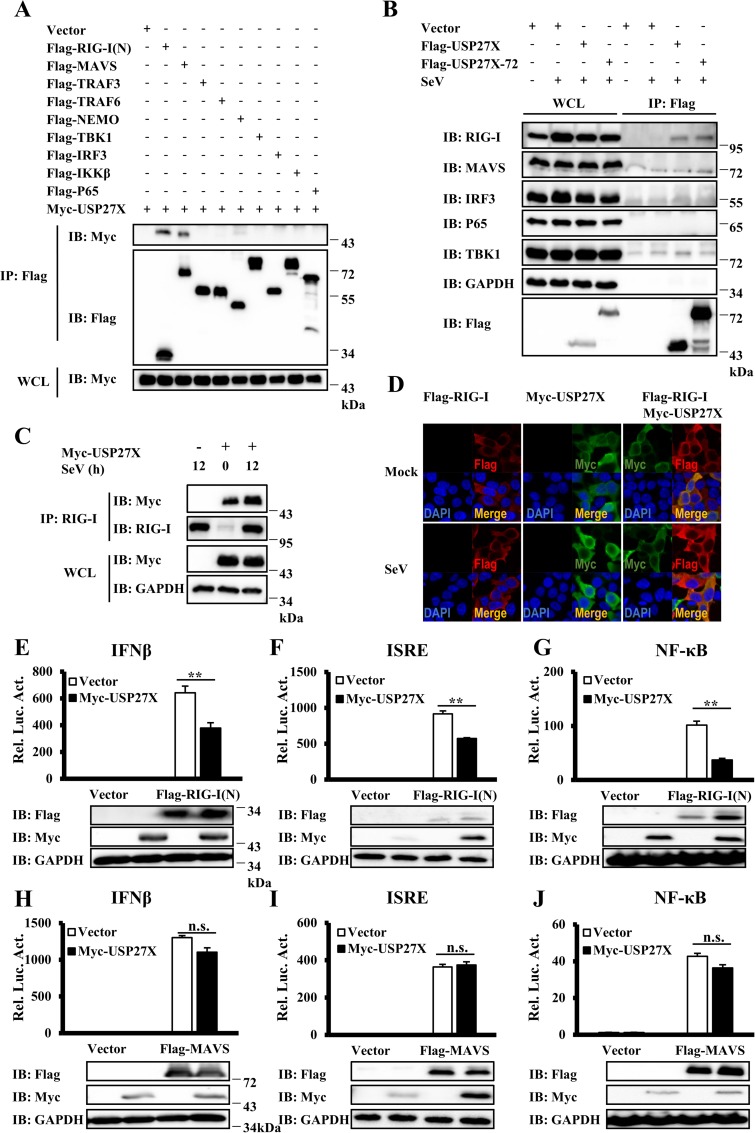
USP27X targets RIG-I to regulate antiviral signaling. (A) HEK293T cells were transfected with the indicated expression plasmids. Twenty-four hours after transfection, the cells were lysed for Co-IP with anti-Flag agarose beads, followed by immunoblotting. The expression levels of transfected proteins in whole cell lysates (WCL) are shown in the bottom panels. (B) HepG2 cells were transfected with Flag-USP27X, or Flag-USP27X-72 expression vector or empty vector. Twenty-four hours after transfection, the cells were mock-infected or infected with SeV for 18 h. Cell lysates were immunoprecipitated with anti-Flag beads, followed by immunoblotting with the indicated antibodies. (C) HEK293T cells were transfected with Myc-USP27X expression vector or empty vector. Twenty-four hours after transfection, the cells were mock-infected or infected with SeV for 12 h. Cell lysates were immunoprecipitated with anti-RIG-I antibody, followed by immunoblotting. (D) HEK293T cells were transfected with the indicated expression plasmids. Twenty-four hours after transfection, cells were mock-infected or infected with SeV (50HA) for 9 h. The cells were fixed, stained with the anti-Flag (red) and anti-Myc (green) antibodies, and observed by confocal microscopy. (E–G) HEK293T cells were co-transfected with the indicated expression plasmids along with luciferase reporter constructs driven by promoters of IFNβ (E), ISRE (F) or NF-κB (G) as well as Renilla as an internal control. Twenty-four hours after transfection, the cells were lysed for luciferase assays (upper panel) and immunoblotting assays (lower panels). (H–J) Similar to (E–G), except that expression plasmids MAVS were used instead of RIG-I(N). The data shown in (E–J) are from one representative experiment of at least three independent experiments (mean ± SD of duplicate experiments). The two-tailed Student’s t-test was used to analyze statistical significance. **P < 0.01; n.s. not significant versus control groups.

The above results indicated that USP27X was involved in regulating type I IFN signaling induced by SeV infection. Since RIG-I-mediated antiviral signaling plays an important role in SeV-induced IFNs production [32], we speculated that USP27X negatively affected IFNs production by regulating RIG-I-mediated signaling. To screen potential target(s) of USP27X, we first performed co-immunoprecipitation (Co-IP) experiments to examine whether USP27X or USP27X-72 interacted with known components of the RIG-I-mediated signaling pathway. As shown in Figs 4A and S9A, the N-terminal domain of RIG-I [RIG-I(N), which contains two CARDs] and MAVS, but not other components, were able to pull down USP27X. Secondly, we examined whether ectopically expressed USP27X could pull down endogenous components of the RIG-I-signaling. Consistently, we detected an interaction between USP27X and RIG-I, but failed to detect the association between USP27X and other components under the same Co-IP conditions (Fig 4B). These results suggested that USP27X regulated SeV-induced IFN signaling by probably targeting RIG-I. In addition, using a RIG-I antibody, we were able to pull down ectopically expressed USP27X and observed that the association between USP27X and RIG-I remarkably increased following Sendai virus infection due to increased RIG-I expression levels (Figs 4C and S9B). Moreover, immunostaining showed significant overlapping signals between overexpressed USP27X and RIG-I in HEK293T cells in the presence or absence of SeV or VSVΔM51-GFP infection (Figs 4D and S9C and S9D).

To further determine the targets of USP27X in RIG-I signaling, we used a reporter assay and found that ectopic expression of USP27X significantly reduced the activation of IFNβ, ISRE and NF-κB induced by RIG-I(N), but not MAVS (Figs 4E–4J and S10A–S10C). Moreover, we also generated RIG-I KO and USP27X/RIG-I double KO cells, and found that loss of RIG-I and USP27X completely failed to induce *IFNB1*, *IFIT1* and *TNFa* mRNA upon SeV infection (S10D–S10F Fig). Of note, there was no apparent difference in levels of TRIM25 expression between WT and USP27X KO cells (S10G and S10H Fig). Taken together, our results suggested that USP27X formed a complex with RIG-I, and primarily targeted RIG-I to regulate innate immune signaling.

### USP27X DUB activity is required for regulation of RIG-I signaling

**Fig 5:**
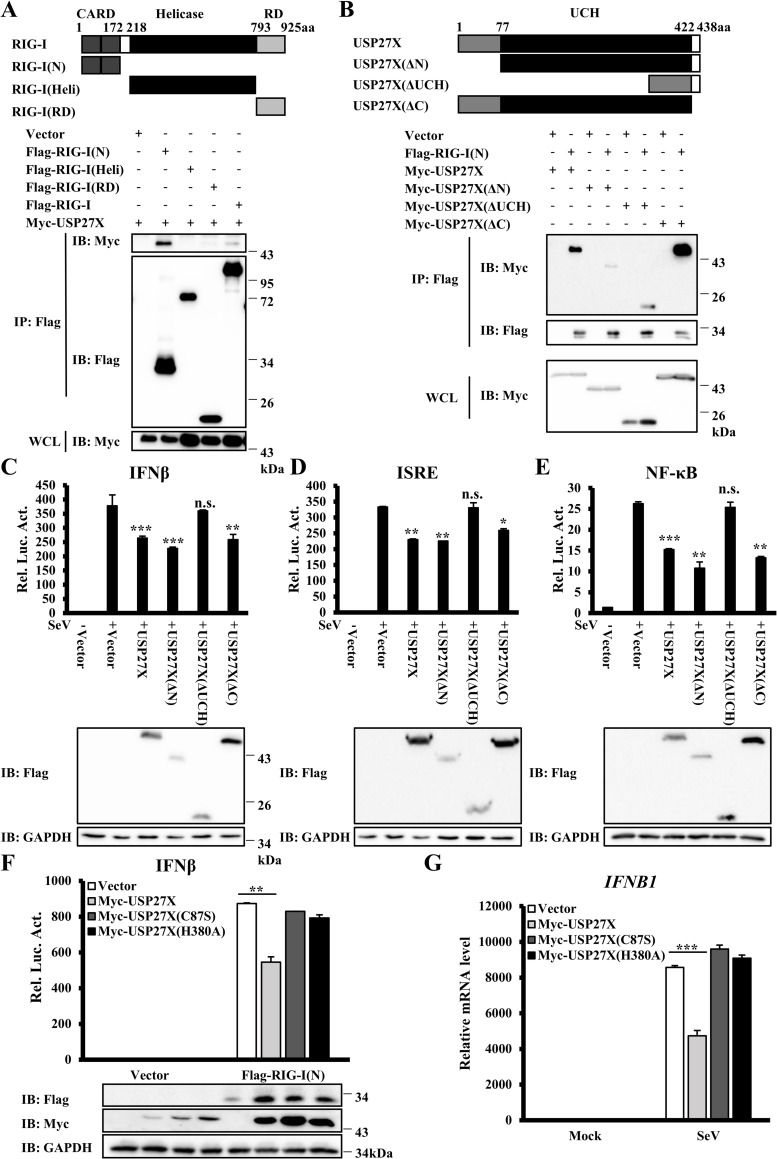
The USP27X UCH domain is essential for its inhibition of type I IFN signaling. (A) HEK293T cells were transfected with the indicated expression plasmids. Cell lysates were immunoprecipitated with anti-Flag beads, followed by immunoblotting. (B) Similar to (A) except that HEK293T cells were transfected with different expression plasmids as indicated. (C–E) HEK293T cells were co-transfected with the indicated expression plasmids along with luciferase reporter constructs driven by promoters of IFNβ (C), ISRE (D) or NF-κB (E). Twenty-four hours after transfection, the cells were infected with SeV for 12 h. The cells were lysed for luciferase assays (upper panel) and immunoblotting assays (lower panels). (F) HEK293T cells were co-transfected with the indicated expression plasmids and IFNβ reporter. Twenty-four hours after transfection, the cells were lysed for luciferase assays (upper panel) and immunoblotting assays (lower panels). (G) HEK293T cells were transfected with the indicated expression plasmids. Twenty-four hours after transfection, the cells were infected with SeV for 9 h. The cells were lysed for measurement of *IFNB1* mRNA levels by qRT-PCR. The data shown in C–G are from one representative experiment of at least three independent experiments [mean ± SD of duplicate experiments in (C–F) or triplicate experiments in (G)]. The two-tailed Student’s t-test was used to analyze statistical significance. * P < 0.05, **P < 0.01, ***P < 0.001, n.s. not significant versus control groups.

To further explore the molecular mechanisms of USP27X in regulation of RIG-I activity, we examined which domain of RIG-I was required for the interaction between RIG-I and USP27X. RIG-I contains two CARDs, a central DExD/H box RNA helicase domain, and a C-terminal RD domain. Co-IP showed that both RIG-I(N) and full-length RIG-I pulled down USP27X, but that the RIG-I helicase domain and RD domain failed to pull down USP27X. These data indicated that USP27X interacted with RIG-I through its CARDs (Figs 5A and S11A).

Next, we mapped which region of USP27X was required for its interaction with RIG-I. Since structural analysis showed that USP27X contained a catalytic UCH domain (residues 78–421), we generated three deletion mutants of USP27X: USP27X-ΔN (deleting residues 1–77), USP27X-ΔUCH (deleting residues 78–421), and USP27X-ΔC (deleting residues 422–438). Co-IP experiments demonstrated that deletion of the UCH domain or the N-terminal region of USP27X significantly inhibited its interaction with RIG-I(N), but the C-terminal region was not required for this interaction (Fig 5B). Reporter assays indicated that loss of the UCH domain, but not the N- or C- terminal regions of USP27X, failed to inhibit activation of IFNβ, ISRE and NF-κB following SeV infection (Fig 5C–5E). These data suggested that the UCH domain of USP27X played an important role in regulating RIG-I signaling. Of note, although the binding of USP27X-ΔN and RIG-I(N) is weaker, USP27X-ΔN still reduced the activation of RIG-I(N), These data suggested that N-terminus 1-77aa of USP27X is dispensable for USP27X to modulate antiviral signaling.

Previous studies revealed that the Cys87 and His380 residues of the USP27X UCH domain were catalytic residues [30, 33], we generated two point mutants (C87S and H380A), which substituted a cysteine residue with serine at position 87 and a histidine residue with alanine at position 380, respectively, to explore whether the function of USP27X in RIG-I signaling was dependent on its DUB enzyme activity. As shown in Fig 5F, both the C87S and H380A mutants failed to inhibit RIG-I(N)-induced IFNβ expression. Consistently, we observed that these catalytically inactive mutants failed to inhibit upregulation of *IFNB1* mRNA following SeV infection (Fig 5G). Moreover, catalytically inactive mutants did not reverse increased levels of *IFNB1* mRNA induced by SeV infection in *USP27X*^*-*/-^ cells (S11B Fig). Taken together, these data indicated that USP27X functioned as a negative regulator of RIG-I signaling in a DUB activity-dependent manner.

### USP27X deubiquitinates the K63-linkage ubiquitination of RIG-I

**Fig 6:**
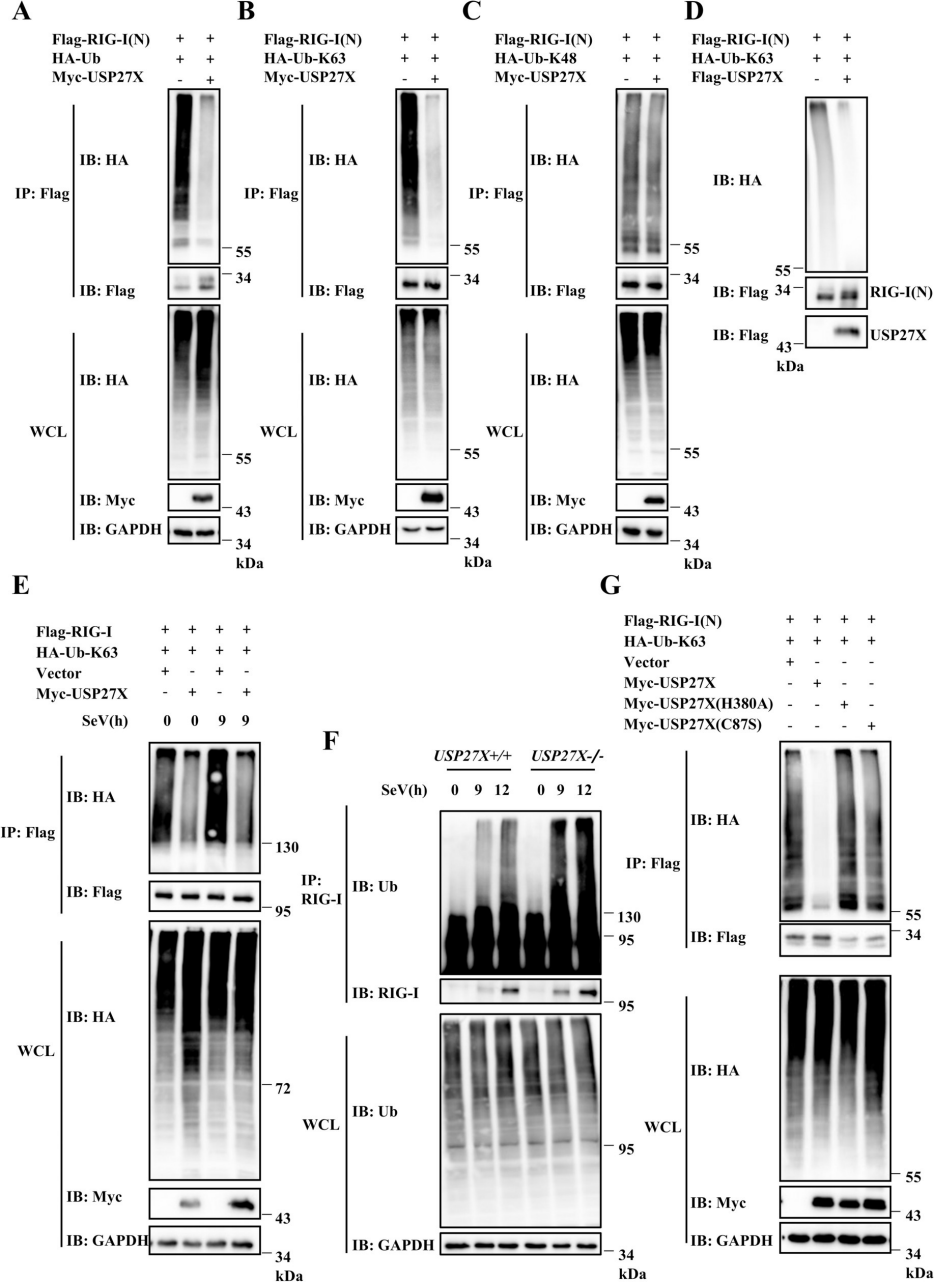
USP27X deubiquitinates the K63-linked ubiquitination of RIG-I. (A–C) HEK293T cells were transfected with USP27X and RIG-I(N) together with HA-tagged wild-type Ub (HA-Ub) (A), HA-Ub-K63 (B), or HA-Ub-K48 (C) plasmids. Twenty-four hours after transfection, cell lysates were immunoprecipitated with anti-Flag beads, followed by immunoblotting analysis with the indicated antibodies. The expression levels of transfected proteins in whole cell lysates (WCL) are shown in the bottom panels. (D) Purified Flag-USP27X and K63-linked ubiquitinated Flag-RIG-I(N) were incubated together in deubiquitination buffer, followed by immunoblotting. (E) HEK293T cells were transfected with the indicated expression plasmids. Twenty-four hours after transfection, cells were infected with SeV for 9 h. Cell lysates were immunoprecipitated with anti-Flag beads and immunoblotted with the indicated antibodies. (F) HEK293T *USP27X*^+/+^ and *USP27X*^-/-^ cells were infected with SeV for 9 or 12 h, lysed, and immunoprecipitated with anti-RIG-I antibody followed by immunoblotting. (G) HEK293T cells were transfected with the indicated expression plasmids. Twenty-four hours after transfection, cell lysates were immunoprecipitated with anti-Flag beads, followed by immunoblotting.

RIG-I ubiquitination plays a pivotal role in its function [10, 18–20]. Since USP27X is a DUB and was associated with RIG-I, our data raised the possibility that USP27X regulated RIG-I-mediated signaling by modulating RIG-I ubiquitination. To test this hypothesis, we conducted *in vivo* ubiquitination assays. As shown in Figs 6A and S12A, overexpression of USP27X markedly decreased levels of RIG-I(N) ubiquitination when HEK293T cells were co-transfected with a vector expressing wild-type ubiquitin. Since Co-IP experiments showed that USP27X was also associated with MAVS, we examined whether USP27X also modulated MAVS ubiquitination. As shown in S12D Fig, we failed to observe an effect of USP27X on MAVS ubiquitination. These data indicated that USP27X specifically attenuated RIG-I, but not MAVS ubiquitination. To determine which type of ubiquitin linkage of polyubiquitin chains on RIG-I(N) is regulated by USP27X, we used ubiquitin mutants in which only one Lysine residue at position 63(K63) or 48 (K48) was available for ubiquitination. As shown in Figs 6B and 6C and S12B and S12C, USP27X overexpression reduced K63-linked ubiquitination of RIG-I(N), but had no effect on K48-linked ubiquitination. To further determine whether USP27X specifically affected K63-linked ubiquitination of RIG-I(N), we pulled down with HA beads (HA-Ub), and probed with Flag antibody for RIG-I(N), and obtain consistent results (S12E Fig). Since K48-linkage ubiquitination of proteins is predominantly involved in protein turnover, we next tested whether USP27X regulated the stability of RIG-I(N). Western blotting confirmed that USP27X had no effect on the expression of RIG-I(N) protein (S13A and S13B Fig). To further examine the function of USP27X in regulating K63-linked ubiquitination of RIG-I(N), we performed *in vitro* ubiquitination assays using purified Flag-tagged USP27X and K63-ubiquitinated RIG-I(N), and obtained results consistent with those of the *in vivo* assays (Fig 6D). Next, we investigated whether USP27X modulated K63-linked ubiquitination of full-length RIG-I. A shown in Fig 6E, USP27X significantly attenuated K63-linked ubiquitination of full-length RIG-I in the presence or absence of SeV infection. Moreover, we explored the function of USP27X in regulating endogenous RIG-I ubiquitination. As shown in Fig 6F, levels of RIG-I ubiquitination induced by SeV infection were notably higher in USP27X-knockout cells than in control cells. In addition, we found that the C87S and H380A mutants of USP27X failed to impair K63-linked ubiquitination of RIG-I(N) (Fig 6G). Collectively, these data suggested that USP27X deubiquitinated K63-linked ubiquitination of RIG-I in a DUB activity-dependent manner, subsequently regulating antiviral signaling.

### USP27X associates with MDA5 and regulates its ubiquitination

**Fig 7:**
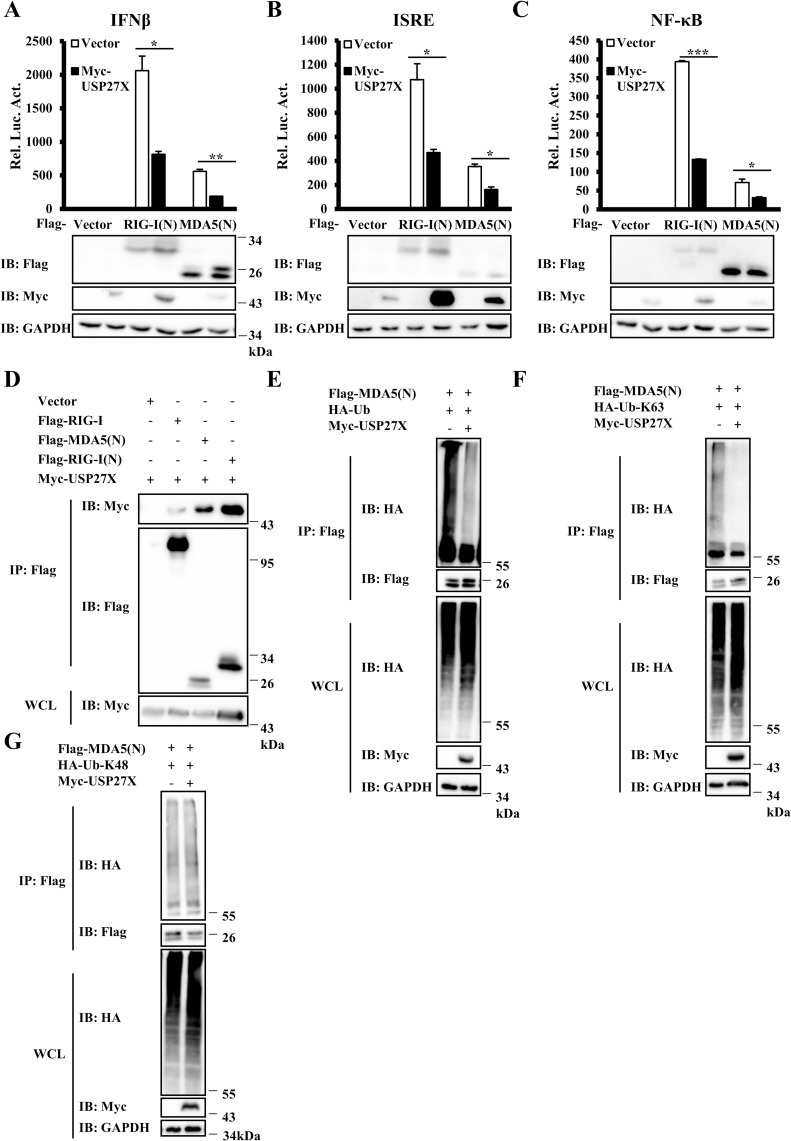
USP27X regulates K63-linked ubiquitination of MDA5. (A–C) HEK293T cells were co-transfected with the indicated expression plasmids, and luciferase reporter constructs driven by promoters of IFNβ (A), ISRE (B) or NF-κB (C). Twenty-four hours after transfection, the cells were lysed for luciferase assays (upper panel) and immunoblotting assays (lower panels). (D) HEK293T cells were transfected with the indicated expression plasmids. Twenty-four hours after transfection, cell lysates were immunoprecipitated with anti-Flag beads, followed by immunoblotting. (E–G) HEK293T cells were transfected with USP27X and MDA5(N) together with HA-tagged wild-type Ub (HA-Ub) (E), HA-Ub-K63 (F), or HA-Ub-K48 (G) plasmids. Twenty-four hours after transfection, cell lysates were immunoprecipitated with anti-Flag beads, followed by immunoblotting analysis with the indicated antibodies. The expression levels of transfected proteins in whole cell lysates (WCL) are shown in the bottom panels. The data shown in A–C are from one representative experiment of at least three independent experiments (mean ± SD of duplicate experiments). The two-tailed Student’s t-test was used to analyze statistical significance. *P < 0.05, **P < 0.01, ***P < 0.001 versus control groups.

MDA5 is another cytosolic receptor involved in sensing RNA virus whose structure is very similar to that of RIG-I [8]. We next considered the possibility that USP27X also played a role in regulating MDA5 function. As shown in Fig 7A–7C, overexpression of USP27X significantly inhibited the activation of IFNβ, ISRE and NF-κB induced by MDA5(N). We next conducted Co-IP experiments and observed that similar to RIG-I(N), MDA5(N) also could form complexes with USP27X (Figs 7D and S14A). We performed *in vivo* ubiquitination assays and found that overexpression of USP27X reduced K63-linked ubiquitination of MDA5(N), but failed to affect K48-linked ubiquitination (Figs 7E–7G and S14B–S14D). Consistently, ubiquitination of MDA5(N) was increased in USP27X KO cells (S14E Fig). Collectively, these results indicated that USP27X modulated MDA5 function by regulating K63-linkage ubiquitination of MDA5.

## Discussion

K63-linked RIG-I ubiquitination is required for its activation. Although several E3 ligases and DUBs have been demonstrated to regulate K63-linked polyubiquitination of RIG-I, the manner in which dynamic ubiquitination and deubiquitination of RIG-I balances antiviral signaling is still unclear. In this study, we identified USP27X as a negative regulator of antiviral signaling through siRNA library screening. Further mechanistic analyses demonstrated that USP27X removed RIG-I K63-linked polyubiquitin chains, subsequently attenuating RIG-I-mediated signaling to maintain innate immune response homeostasis.

USP27X is a DUB and member of the cysteine protease family. Previous studies indicated that USP27X is involved in several biological processes through removal of K48-linked polyubiquitin chains regulating protein degradation [28–30]. In this study, we demonstrated that USP27X targeted RIG-I by removing its K63-linked polyubiquitin chains. We found that overexpression of USP27X reduced type I IFN signaling induced by the constitutively active form of RIG-I [RIG-I(N)] or by SeV infection, whereas USP27X knockdown or knockout significantly enhanced SeV-induced type I IFN signaling in a variety of cell lines. Of note, by measuring levels of *USP27X* transcripts in multiple time points after viral infection, we found that expression of USP27X displayed a constant pattern, at least, at transcriptional levels (S15A and S15B Fig). Since expression of RIG-I, but not USP27X, is viral inducible, our findings suggested that USP27X plays an important role in constitutively suppressing the RIG-I signaling. Importantly, we found that while ectopic expression of USP27X reduced K63-linked polyubiquitination of RIG-I(N) in a manner dependent on its DUB activity, USP27X knockout augmented K63-linked polyubiquitination of RIG-I. It is worth to note that 172 lysine residue has been shown as one of the major modified site of RIG-I through K63-linked ubiquitination[10], our findings suggest that alteration of USP27X expression affected levels of K63-linked ubiquitination of RIG-I(N), even RIG-I(N)-K172R form, in which the 172 lysine residue was replaced with arginine (R) (S16 Fig). These findings suggest that USP27X removes K63-linked ubiquitination of RIG-I likely at multiple sites including the 172 lysine residue.

Previous studies have reported that USP27X could be translated from an upstream translation start site (CTG), this product therefore contained an additional 198 aa fragment at its N-terminus [28, 30]. Although we tried our best to generate the antibodies specifically against the endogenous USP27X translational products, we failed to obtain the suitable antibodies including those from commercially available sources, thus we were not able to determine which form of USP27X existing in cells (S17 Fig). Nevertheless, by performing functional analysis using both USP27X and USP27X-72, we found that both USP27X and USP27X-72 inhibited the activation of IFNβ, ISRE and NF-κB by overexpression of RIG-I(N) or upon SeV infection. Moreover, our ubiquitination assays suggest that USP27X-72 deubiquitinated K63-linked ubiquitination of RIG-I(N) and MDA5(N) (S12B and S14C Figs). Importantly, rescue experiments suggest that similar to USP27X, USP27X-72 could reverse the enhanced antiviral responses in USP27X knockout cells (S11B Fig). Additionally, overexpression of 1–198 aa fragment of the USP27X-72 had no role in affecting activation of IFNβ, ISRE and NF-κB upon SeV infection (S3B–S3D Fig), and ubiquitination levels of RIG-I(N) and MDA5(N) (S18A and S18B Fig). Thus, our findings suggest that 1–198 aa in the N-terminus of USP27X-72 was dispensable for USP27X to modulate antiviral signaling. Taken together, these results suggest that both USP27X-72 and USP27X display important roles in regulating RIG-I-mediated signaling.

A number of studies including ours indicated that K63-linked of RIG-I ubiquitination is regulated by several DUBs, including USP3, USP21, USP25, USP15 and CYLD [24, 26, 34–37]. Similar to USP27X, USP3 also deubiquitinated MDA5, whereas whether USP21 and USP25 are involved in regulating MDA5 ubiquitination remains unclear. Of note, USP15 was also found to regulate K63-linked polyubiquitination of RIG-I. However, unlike other RIG-I DUBs, the function of USP15 was not dependent on its DUB activity, suggesting that other regulatory mechanisms may be involved. These data suggested that there are several DUBs involved in regulating K63-linked polyubiquitination of RIG-I. However, the roles of different DUBs in regulating the function of RIG-I through removing K63-linkage polyubiquitination remain elusive. To address this issue, we measured mRNA levels of a number of DUBs (including USP27X, USP3, USP21 and CYLD) that have been reported to target RIG-I in multiple cell lines, and found that the mRNAs of these DUBs displayed distinguish expression patterns in different cell lines (S5A and S19A–S19C Figs). To distinguish the roles of different DUBs in RIG-I-mediated pathway, we have generated these DUBs KO mutants in HEK293T cells, since these DUBs all express in HEK293T cells. Using these DUBs KO cells, we found that individual loss of these DUBs all led to up-regulation of antiviral signaling. Of note, USP27X exhibited the highest activity in antagonizing RIG-I signaling in HEK293T cells, suggesting that USP27X might function as one of major DUBs in maintaining the RIG-I signaling in HEK293T cells (S19D and S19E Fig). Taken together, our findings suggest that homeostasis of the RIG-I-mediated antiviral signaling involves multiple DUBs, the specific roles of different DUBs in inhibiting RIG-I likely depend on their scheduled expression in certain cell types.

Collectively, our studies uncovered a novel role of USP27X in regulating antiviral signaling. USP27X interacted with RIG-I and MDA5, and removed K63-linked polyubiquitin chains from RIG-I and MDA5 to suppress the excessive production of type I IFNs. Our data provide insights into the mechanisms through which USP27X functions as a negative regulator of antiviral signaling to maintain innate immune signaling homeostasis.

## Materials and methods

### Cell culture

HEK293T, HeLa, HepG2 and RAW264.7 cells were obtained from the Shanghai Cell Bank of the Chinese Academy of Sciences, and maintained in Dulbecco’s modified Eagle’s medium (Invitrogen) containing 10% (v/v) fetal bovine serum (Invitrogen) and 1% streptomycin and penicillin. THP-1 cells were cultured in RPMI-1640 containing 10% fetal bovine serum, 1% streptomycin and penicillin and 10 μM β-mercaptoethanol.

### Antibodies

Rabbit anti-p-IRF3, anti-IRF3, and anti-p-P65 antibodies were from Cell Signaling Technology. Mouse anti-P65 and TRIM25 antibodies were from Santa Cruz. Rabbit anti-Flag antibody was from Sigma. Rabbit anti-Myc, anti-Sendai pAb and anti-HA antibodies were from MBL. Mouse anti-glyceraldehyde-3-phosphate dehydrogenase (GAPDH) and mouse anti-HA antibodies were from Sungene Biotechnology.

### Plasmids

Flag-tagged RIG-I, RIG-I(N), RIG-I(Heli), RIG-I(RD), USP27X, USP27X-72, MAVS, TRAF3, TRAF6, NEMO, TBK1, IRF3, IKKβ and P65, Myc-tagged USP27X, USP27X-72, USP27X-72(N) and HA-tagged Ub, Ub-K63 and Ub-K48 were constructed using standard molecular biology methods. USP27X mutants were generated by PCR using Pfu DNA polymerase. The plasmids encoding IFNβ-Luc, NF-κB-Luc and ISRE-Luc have been described previously [37].

### Transfection and luciferase reporter assay

HEK293T cells were seeded in 24-well plates and cultured overnight, then transfected with plasmids using polyethylenimine. A Renilla reporter plasmid and a firefly luciferase reporter plasmid encoding IFNβ-Luc, NF-κB-Luc or ISRE-Luc were transfected along with the indicated expression plasmids. In the same experiment, empty control plasmid was added to ensure that the same amount of total DNA was transfected. Twenty-four hours following transfection, cells were lysed to measure luciferase activity, which was normalized to Renilla activity. All reporter assays were repeated at least three times.

### Co-IP and immunoblotting analysis

Cells were lysed in lysis buffer (0.5% Triton X-100, 20 mM Tris-HCl pH 7.5, 150 mM NaCl, 10% glycerol, 1 mM EDTA). Clarified cell lysates were incubated with anti-Flag agarose beads (Sigma) for 4 h. The immunoprecipitated complexes were washed with lysis buffer containing 300 mM NaCl three times and subjected to immunoblotting analysis with the indicated antibodies. For semi-endogenous IP, cell lysates were incubated with RIG-I antibody overnight at 4°C, followed by further incubation with protein A/G beads (Pierce) for 2 h. Immunoblotting was carried out using standard procedures.

### Immunofluorescence assays

HEK293T and HepG2 cells were grown on gelatin-coated glass coverslips, and transfected or infected by SeV as indicated. Cells were washed with phosphate-buffered saline, fixed with 4% paraformaldehyde for 15 min, permeabilized with 0.2% Triton X-100 for 10 min, and then blocked with 5% (w/v) bovine serum albumin for 30 min. Cells were then incubated with primary and secondary antibodies. Imaging was conducted using a Zeiss LSM 710 META laser scanning confocal system.

### *In vivo* and *in vitro* deubiquitination assays

For *in vivo* deubiquitination assays, HEK293T cells were transfected with the indicated plasmids. Cells were treated with MG132 (final concentration 25 μM) before harvesting. Cells were lysed in lysis buffer A (150 mM NaCl, 50 mM Tris-HCl pH 7.5, 0.5% Nonidet P-40, 10% glycerol, 1% SDS, 1 mM EDTA). The supernatants were denatured at 95°C for 5 min, diluted in buffer B (150 mM NaCl, 50 mM Tris-HCl pH 7.5, 0.5% Nonidet P-40, 10% glycerol, 1 mM EDTA) until the concentration of SDS was about 0.1%, then immunoprecipitated with anti-Flag beads for 4 h. After washing with wash buffer C (500 mM NaCl, 50 mM Tris-HCl pH 7.5, 0.5% Nonidet P-40, 10% glycerol, 1 mM EDTA) three times, the immunoprecipitated material was subjected to western blotting with the indicated antibodies.

For *in vitro* deubiquitination assays, HEK293T cells were co-transfected with plasmids encoding Flag-RIG-I(N) and HA-Ub-K63. Cells were lysed with lysis buffer (0.5% Triton X-100, 20 mM Tris-HCl pH 7.5, 150 mM NaCl, 10% glycerol, 1 mM EDTA) and ubiquitinated RIG-I(N) was purified using anti-Flag beads. Ubiquitinated RIG-I(N) proteins were eluted with Flag peptide after extensive washing with lysis buffer (0.5% Triton X-100, 20 mM Tris-HCl pH 7.5, 300 mM NaCl, 10% glycerol, 1 mM EDTA). Flag-tagged USP27X proteins were purified using the same method. Ubiquitinated RIG-I(N) was incubated with USP27X in deubiquitination buffer (50 mM Tris-HCl pH 8.0, 50 mM NaCl, 5% glycerol, 1 mM EDTA, 1 mM ATP) at 37°C for 2 h, then incubated at 16°C overnight, followed by immunoblotting assays.

### Viral plaque assay

HEK293T *USP27X* ^+/+^ and *USP27X*^-/-^ cells were infected with VSVΔM51-GFP at a multiplicity of infection (MOI) of 0.01 for 9 h, and culture supernatants were collected and diluted to infect Vero cells. Thirty-six hours later, Vero cells were fixed with methanol for 30 min and stained with 1% crystal violet. Plaques were counted to quantitate viral titer and shown in plaque-forming units (p.f.u.)/mL.

### RNAi screening

A genome-wide siRNA screen using the Silencer Human Ubiquitin siRNA Library (Dharmacon) was conducted by transfection a HEK293T stable reporter cell line that expresses firefly luciferase driven by a human IFNβ promoter. Forty-eight hours later, cells were infected with SeV, followed by reporter assays.

### Lentivirus-mediated knockdown of USP27X

RAW264.7 cells were infected with lentiviral vectors targeting two different regions of mUsp27x (shRNA1, 2) or empty vector for 48 h. The cells were either untreated or infected with SeV or VSVΔM51-GFP for the indicated times, followed by subsequent experiments. Knockdown efficiency was determined by qRT-PCR. The sequences for mouse Usp27x shRNAs were as follows (5’–3’): shUsp27x1: TCATGTGCCCTATAAGTTA; shUsp27x2: GCGCAAGATCACTACGTACAT. The sequence for human USP27X shRNA was (5’–3’): AAACAACCAAACCAGAATTA.

### qRT–PCR

Total RNA was extracted from cells using TRIZOL reagent (Invitrogen) and cDNA was synthesized using the SuperScript III First-Strand cDNA Synthesis kit (Invitrogen). qRT-PCRs were conducted using SYBR Green Master Mix (Thermo Fisher) and a Light Cycler 480 instrument (Roche). The 2^-ΔΔCt^ method was used to calculate relative gene expression, and relative mRNA level for each gene was normalized to levels of GAPDH. Data shown are the relative abundance of mRNA as compared with control groups. All samples were assayed in triplicate. The gene-specific primers were as follows (5’–3’):

hUSP27X-S CAACCTGGGAAACAACCAAACC;

hUSP27X-AS GGCCTGGACAATGCAGTTCAT;

hIFNB1-S AGGACAGGATGAACTTTGAC;

hIFNB1-AS TGATAGACATTAGCCAGGAG;

hIFIT1-S TACCTGGACAAGGTGGAGAA;

hIFIT1-AS GTGAGGACATGTTGGCTAGA;

hIL6-S TCCAGAACAGATTTGAGAGTAGTG;

hIL6-AS GCATTTGTGGTTGGGTCAGG;

hTNFα-S CTGCCCCAATCCCTTTATT;

hTNFα-AS CCCAATTCTCTTTTTGAGCC;

hGAPDH-S ATGACATCAAGAAGGTGGTG;

hGAPDH-AS CATACCAGGAAATGAGCTTG;

hUSP3-S GAGCTGCCTGTATGACCT;

hUSP3-AS ACCACAGTCTCCTCGTCA;

hUSP21-S ACCGAGCCAACCTAATGTG;

hUSP21-AS GGCAGGGACAGGTCACAA;

hCYLD-S CATAATAAACCAAAGGCTAC;

hCYLD-AS GTGAAGAACGGTCAAAGT;

hTRIM25-S AGCAGCTACAACAAGAATACACG;

hTRIM25-AS GGCTCTGTTCAATCTCCTCCT;

mGapdh-S AACTTTGGCATTGTGGAAGG;

mGapdh-AS ACACATTGGGGGTAGGAACA;

mIfnb1-S ATGGTGGTCCGAGCAGAGAT;

mIfnb1-AS CCACCACTCATTCTGAGGCA;

mIfit1-S CTGAGATGTCACTTCACATGGAA;

mIfit1-AS GTGCATCCCCAATGGGTTCT;

mIl6-S TAGTCCTTCCTACCCCAATTTCC;

mIl6-AS TTGGTCCTTAGCCACTCCTTC;

mUsp27x-S CCAAAGAAGAGCAAGGAG;

mUsp27x-AS GTAAGCCGATGGTAAAGC.

### CRISPR/Cas9-mediated USP27X knockout cell lines

To generate stable USP27X knockout cells (USP27X-KO) including HEK293T, HeLa, RAW 264.7 and L929 cells, RIG-I-knockout, and USP27X and RIG-I double-knockout HEK293T cell line, cells were transfected or infected (RAW 264.7 and L929 cells) with the lentiCRISPRv2, lentiCRISPRv2-USP27X or lentiCRISPRv2-RIG-I vectors. lentiCRISPRv2-USP27X or RIG-I vectors were constructed according to the method described by Sanjana *et al* [38]. USP27X-knockout cells were verified by DNA sequencing (S20 Fig). gRNA sequences were as follows (5’-3’):

hUSP27X-gRNA1: TCTTAAACCGATCGTAAAGC;

hUSP27X-gRNA2: ATCTCTCAGTATCGGCGTGT;

mUsp27x-gRNA1: AATTAGTCCTCGTAAGCCGA;

mUsp27x-gRNA2: ACAAGCGTCCACCTCGACAG;

hRIG-I-gRNA: ACATCCTGAGCTACATGGCCC.

To generate stable USP27X-knockout HepG2 cell line, USP27X, USP21, USP3-knockout HEK293T cell lines, cells were transfected with the pX330-GFP or pX330-GFP-USP27X/USP21/USP3 vectors. Knockout cells were verified by DNA sequencing. gRNA sequences were as follows (5’-3’):

hUSP27X-gRNA1: GTGACAGGCCATCATTTCGG;

hUSP27X-gRNA2: GGGAAGCCTGCATAGACGAG;

hUSP27X-gRNA3: GAGCAGGAAAATGCTGACAC;

hUSP27X-gRNA4: ACCTGGGCTAGTGAAAGGAG;

hUSP21-gRNA1: GGGAGCAGTATACGCACTGA;

hUSP21-gRNA2: GTGGGGACTTGCGTCCAATG;

hUSP21-gRNA3: GGTATACAGGACTTCCTGCG;

hUSP21-gRNA4: TTCGAGTTTTCTGCCGACAT;

hUSP3-gRNA1: AGGTTATCTACATTGGCGTG;

hUSP3-gRNA2: GTGGTTACTATGATCCTGTG;

hUSP3-gRNA3: TCTGGACATCTTCCACACAA;

hUSP3-gRNA4: ATGTTAACTGGACCAGGGAC.

### Statistical analysis

All statistical data are presented as means ± standard deviations (SDs) and the Student’s t-test was used for all statistical analyses. For all tests, a p value of less than 0.05 was considered statistically significant.

### Accession numbers

The mRNA sequence data for genes described in this study can be found in the NCBI under the following accession numbers: Homo sapiens USP27X (NM_001145073.2), Homo sapiens RIG-I (NM_014314.4), MDA5 (NM_022168.4), Homo sapiens MAVS (NM_020746.5) and Mus musculus Usp27x(NM_019461.4).

## Supporting information

S1 Fig Identification of USP27X as a negative regulator of type I IFN signaling. HEK293T stable reporter cell line expressing firefly luciferase driven by a human IFNβ promoter was transfected with the indicated siRNAs. Forty-eight hours after transfection, cells were infected with SeV for 12 h, followed by luciferase assays.
(TIF)

S2 Fig Overexpression of USP27X reduces nuclear translocation of IRF3 and P65 upon SeV infection. HEK293T cells were transfected with the indicated expression plasmids. Twenty-four hours after transfection, cells were mock-infected or infected with SeV (50HA) for 9 h. The cells were fixed, stained with the anti-IRF3 (red), anti-P65 (red) and anti-Myc (green) antibodies, and observed by confocal microscopy.
(TIF)

S3 Fig USP27X-72 overexpression inhibits type I IFN signaling. (A) Schematic diagram of USP27X-72. (B–D) HEK293T cells were co-transfected with the indicated expression plasmids along with luciferase reporter constructs driven by promoters of IFNβ (B), ISRE (C) or NF-κB (D). Twenty-four hours after transfection, the cells were infected with SeV for 12 h. The cells were lysed for luciferase assays (upper panel) and immunoblotting assays (lower panels). The data shown in (B–D) are from one representative experiment of at least three independent experiments (mean ± SD of duplicate experiments). The two-tailed Student’s t-test was used to analyze statistical significance. *P < 0.05; n.s. not significant versus control groups.
(TIF)

S4 Fig USP27X is not involving in regulating TLR3/4-mediated IFN signaling in RAW 264.7 cells. RAW264.7 cells were infected with lentiviral vectors targeting Usp27x (shUsp27x1) or empty vector for 48 h, followed by stimulation with Poly(I:C) or LPS for the indicated times. The cells were lysed for immunoblotting with the indicated antibodies.
(TIF)

S5 Fig Knockdown of USP27X increases type I IFN signaling in HepG2 cells. (A) qRT-PCR assays were performed to measure levels of *USP27X* mRNA in a number of cell lines. (B–E) HepG2 cells were infected with lentiviral vectors targeting USP27X (shUSP27X) or empty vector for 48 h, followed by SeV infection for 12 h. The cells were collected for qRT-PCR assays to measure mRNA levels of *USP27X* (B), *IFNB1* (C), *TNFα* (D) and *IFIT1* (E). The data shown in (A–E) are from one representative experiment of at least three independent experiments (mean ± SD of triplicate experiments). The two-tailed Student’s t-test was used to analyze statistical significance. ***P < 0.001 versus control groups.
(TIF)

S6 Fig Knockout of USP27X enhances type I IFN signaling. (A–B) HeLa (A) or HepG2 (B) *USP27X*^*+/+*^ and *USP27X*^*-/-*^ cells were infected with SeV for 9 h or transfected with Poly(I:C) for 6 h, then lysed for measurement of *IFNB1*, *IFIT1*, and *IL6* or *TNFα* mRNA levels by qRT-PCR. (C) L929 *Usp27x*^*+/+*^ and *Usp27x*^*-/-*^ cells were infected with SeV for the indicated times, then lysed for measurement of *Ifnb1*, *Ifit1* and *Il6* mRNA levels by qRT-PCR. (D) RAW264.7 *Usp27x*^+/+^ and *Usp27x*^-/-^ cells were infected with SeV for 6 h, then lysed for measurement of *Ifnb1*, *Ifit1* and *Il6* mRNA levels by qRT-PCR. The data shown in (A–D) are from one representative experiment of at least three independent experiments (mean ± SD of triplicate experiments). The two-tailed Student’s t-test was used to analyze statistical significance. *** P < 0.001 versus control groups.
(TIF)

S7 Fig Knockout of USP27X enhances nuclear translocation of IRF3 and P65 upon SeV infection. HepG2 *USP27X*^*+/+*^ and *USP27X*^*-/-*^ cells were mock-infected or infected with SeV (100HA) for 9 h. The cells were fixed, stained with the anti-IRF3 (red) (left panels) or anti-P65 (red) (right panels) antibodies, and observed by confocal microscopy.
(TIF)

S8 Fig USP27X is involved in regulating viral amplification in HepG2 cells. HepG2 *USP27X* ^*+/+*^ and *USP27X*^*-/-*^ cells were infected with VSVΔM51-GFP at an MOI of 0.01 for 12 h. Culture supernatants were collected to measure viral titers by plaque assay. The data shown in the right panel are from one representative experiment of at least three independent experiments (mean ± SD duplicate experiments). The two-tailed Student’s t-test was used to analyze statistical significance. *** P < 0.001 versus control groups.
(TIF)

S9 Fig USP27X interacts with RIG-I. (A) HEK293T cells were transfected with the indicated expression plasmids. Twenty-four hours after transfection, the cells were lysed for Co-IP with anti-Flag agarose beads, followed by immunoblotting. The expression levels of transfected proteins in whole cell lysates (WCL) are shown in the bottom panels. (B) HEK293T cells were transfected with Myc-USP27X-72 expression vector or empty vector. Twenty-four hours after transfection, the cells were mock-infected or infected with SeV for 12 h. Cell lysates were immunoprecipitated with anti-RIG-I antibody, followed by immunoblotting. (C) HEK293T cells were transfected with the indicated expression plasmids. Twenty-four hours after transfection, cells were mock-infected or infected with SeV (50HA) for 9 h. The cells were fixed, stained with the anti-Flag (red) and anti-Myc (green) antibodies, and observed by confocal microscopy. (D) HEK293T cells were transfected with the indicated expression plasmids. Twenty-four hours after transfection, cells were mock-infected or infected with VSVΔM51-GFP (1 MOI) for 9 h. The cells were fixed, stained with the anti-Flag (red) and anti-Myc (pink) antibodies, and observed by confocal microscopy.
(TIF)

S10 Fig USP27X targets RIG-I to negatively regulate antiviral signaling. (A–C) HEK293T cells were co-transfected with the indicated expression plasmids along with luciferase reporter constructs driven by promoters of IFNβ (A), ISRE (B) or NF-κB (C) as well as Renilla as an internal control. Twenty-four hours after transfection, the cells were lysed for luciferase assays (upper panel) and immunoblotting assays (lower panels). (D–G) HEK293T *WT*, *USP27X*^*-/-*^, *RIG-I*^*-/-*^ and *USP27X/RIG-I*^*-/-*^ cells were infected with SeV for 9 h, then lysed for measurement of *IFNB1* (D), *IFIT1* (E), *TNFα* (F) and *TRIM25* (G) mRNA levels by qRT-PCR. (H) HeLa *USP27X*^*+/+*^ and *USP27X*^-/-^ cells were infected with SeV for the indicated times, then lysed for immunoblotting with the indicated antibodies. The data shown in (A–G) are from one representative experiment of at least three independent experiments [mean ± SD of duplicate experiments in (A–C) or triplicate experiments in (D–G)]. The two-tailed Student’s t-test was used to analyze statistical significance. * P < 0.05; **P < 0.01; ***P < 0.001; n.s. not significant versus control groups.
(TIF)

S11 Fig USP27X interacts with RIG-I through RIG-I CARDs and requires its DUB activity for regulation of RIG-I signaling. (A) HEK293T cells were transfected with the indicated expression plasmids. Cell lysates were immunoprecipitated with anti-Flag beads, followed by immunoblotting. (B) HEK293T *USP27X*^*+/+*^ and *USP27X*^*-/-*^ cells were transfected with indicated expression plasmids. Twenty-four hours after transfection, the cells were infected with SeV for 9 h, followed by measurement of *IFNB1* mRNA levels by qRT-PCR. The data shown in (B) are from one representative experiment of at least three independent experiments (mean ± SD of triplicate experiments). The two-tailed Student’s t-test was used to analyze statistical significance. *** P < 0.001 versus control groups.
(TIF)

S12 Fig USP27X deubiquitinates the K63-linked ubiquitination of RIG-I. (A–E) HEK293T cells were transfected with the indicated plasmids. Cell lysates were immunoprecipitated with anti-Flag or anti-HA beads, followed by immunoblotting with the indicated antibodies.
(TIF)

S13 Fig USP27X does not affect RIG-I(N) stability. (A) HEK293T cells were transfected with the indicated plasmids. Twenty-four hours after transfection, the cells were lysed for immunoblotting with the indicated antibodies. (B) HEK293T *USP27X*^*+/+*^ and *USP27X*^*-/-*^ cells were co-transfected with Myc-GFP and Flag-RIG-I(N) plasmids. Twenty-four hours after transfection, cell lysates were for immunoblotting analysis with the indicated antibodies.
(TIF)

S14 Fig USP27X regulates K63-linked ubiquitination of MDA5. (A) HEK293T cells were transfected with the indicated expression plasmids. Cell lysates were immunoprecipitated with anti-Flag beads, followed by immunoblotting. (B–D) HEK293T cells were transfected with USP27X-72 and MDA5(N) together with HA-tagged wild-type Ub (HA-Ub) (B), HA-Ub-K63 (C), or HA-Ub-K48 (D) plasmids. Twenty-four hours after transfection, cell lysates were immunoprecipitated with anti-Flag beads, followed by immunoblotting analysis with the indicated antibodies. The expression levels of transfected proteins in whole cell lysates (WCL) are shown in the bottom panels. (E) HepG2 *USP27X*^*+/+*^ and *USP27X*^*-/-*^ cells were transfected with Flag-MDA5(N) plasmids. Twenty-four hours after transfection, cell lysates were immunoprecipitated with anti-Flag beads, followed by immunoblotting analysis with the indicated antibodies. The expression levels of transfected proteins in whole cell lysates (WCL) are shown in the bottom panels.
(TIF)

S15 Fig *USP27X* mRNA is not induced by SeV infection. HEK293T (A) or HepG2 (B) were infected with SeV for the indicated times, then lysed for measurement of *USP27X* and *IFNB1* mRNA levels by qRT-PCR. The data shown in (A–B) are from one representative experiment of at least three independent experiments (mean ± SD of triplicate experiments). The two-tailed Student’s t-test was used to analyze statistical significance. ***P < 0.001, versus control groups.
(TIF)

S16 Fig K63-linked ubiquitination of RIG-I(N) and RIG-I(N)-K172R mutant are both increased in USP27X KO cells. HepG2 *USP27X*^*+/+*^ and *USP27X*^*-/-*^ cells were transfected with Flag-RIG-I(N) or Flag-RIG-I(N)-K172R plasmids. Twenty-four hours after transfection, cell lysates were immunoprecipitated with anti-Flag beads, followed by immunoblotting analysis with the indicated antibodies. The expression levels of transfected proteins in whole cell lysates (WCL) are shown in the bottom panels.
(TIF)

S17 Fig USP27X antibodies were tested in *USP27X*^+/+^ and *USP27X*^-/-^ cells. (A) *USP27X*^+/+^ and *USP27X*^-/-^ cells including HEK293T, HeLa and HepG2 cells were lysed for immunoblotting with the indicated antibodies. (B) *Usp27x*^+/+^ and *Usp27x*^-/-^ cells including L929 and RAW264.7 cells were lysed for immunoblotting with the indicated antibodies.
(TIF)

S18 Fig 1–198 aa in the N-terminus of USP27X-72 is dispensable for USP27X to modulate antiviral signaling. (A–B) HEK293T cells were transfected with USP27X, USP27X-72(N) and RIG-I(N) (A) or MDA5(N) (B) together with HA-tagged Ub-K63 plasmids. Twenty-four hours after transfection, cell lysates were immunoprecipitated with anti-Flag beads, followed by immunoblotting analysis with the indicated antibodies. The expression levels of transfected proteins in whole cell lysates (WCL) are shown in the bottom panels.
(TIF)

S19 Fig Knockout of USP27X, USP3, USP21 and CYLD enhance type I IFN signaling. (A–C) qRT-PCR assays were performed to measure levels of *USP3* (A), *USP21* (B) and *CYLD* (C) mRNA in a number of cell lines. (D) HEK293T *WT*, *USP27X*^*-/-*^, *USP3*^*-/-*^ and *USP21*^*-/-*^ cells were infected with SeV for 9 h, then lysed for measurement of *IFNB1* mRNA levels by qRT-PCR. (E) HEK293T *WT* and *CYLD*^*-/-*^ cells were infected with SeV for the indicated times, then lysed for measurement of *IFNB1* mRNA levels by qRT-PCR. The data shown in (A–E) are from one representative experiment of at least three independent experiments (mean ± SD of triplicate experiments). The two-tailed Student’s t-test was used to analyze statistical significance. * P < 0.05; **P < 0.01; ***P < 0.001, versus control groups.
(TIF)

S20 Fig Validation of USP27X-KO cells by sequencing. USP27X-KO cells including HEK293T, HeLa, HepG2, L929 and RAW264.7 cells were generated by CRISPR/Cas9 gene editing system, and USP27X-KO cells were verified by DNA sequencing.
(TIF)
