# Supplementary figures and images for: USP27X negatively regulates antiviral signaling by deubiquitinating RIG-I
Source: PLoS Pathog. 2020 Feb 6;16(2):e1008293. doi: 10.1371/journal.ppat.1008293 (PMC7029883; doi:10.1371/journal.ppat.1008293)

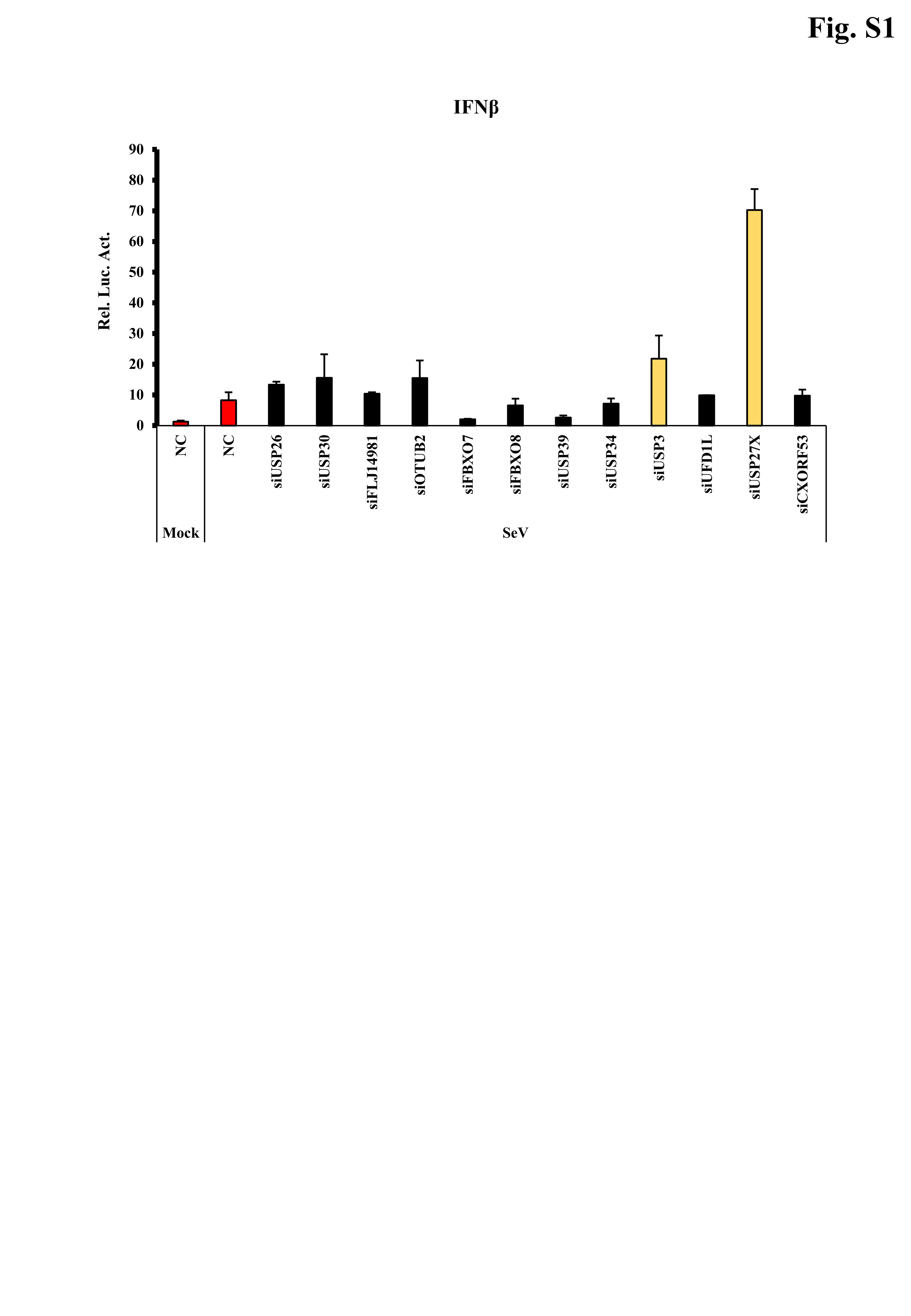

Supplement: S1 Fig — HEK293T stable reporter cell line expressing firefly luciferase driven by a human IFNβ promoter was transfected with the indicated siRNAs. Forty-eight hours after transfection, cells were infected with SeV for 12 h, followed by luciferase assays. (TIF) [file ppat.1008293.s001.tif]

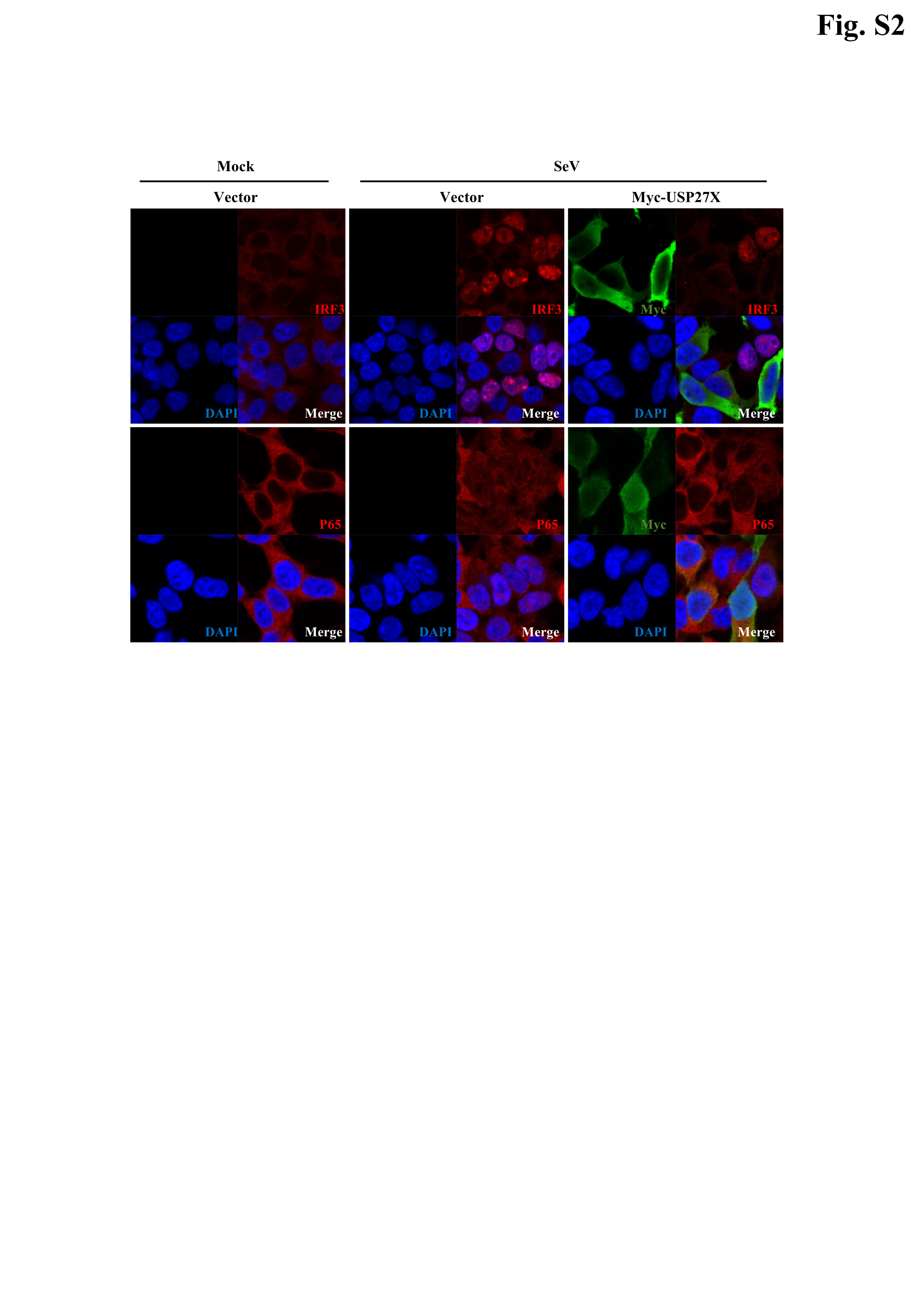

Supplement: S2 Fig — HEK293T cells were transfected with the indicated expression plasmids. Twenty-four hours after transfection, cells were mock-infected or infected with SeV (50HA) for 9 h. The cells were fixed, stained with the anti-IRF3 (red), anti-P65 (red) and anti-Myc (green) antibodies, and observed by confocal microscopy. (TIF) [file ppat.1008293.s002.tif]

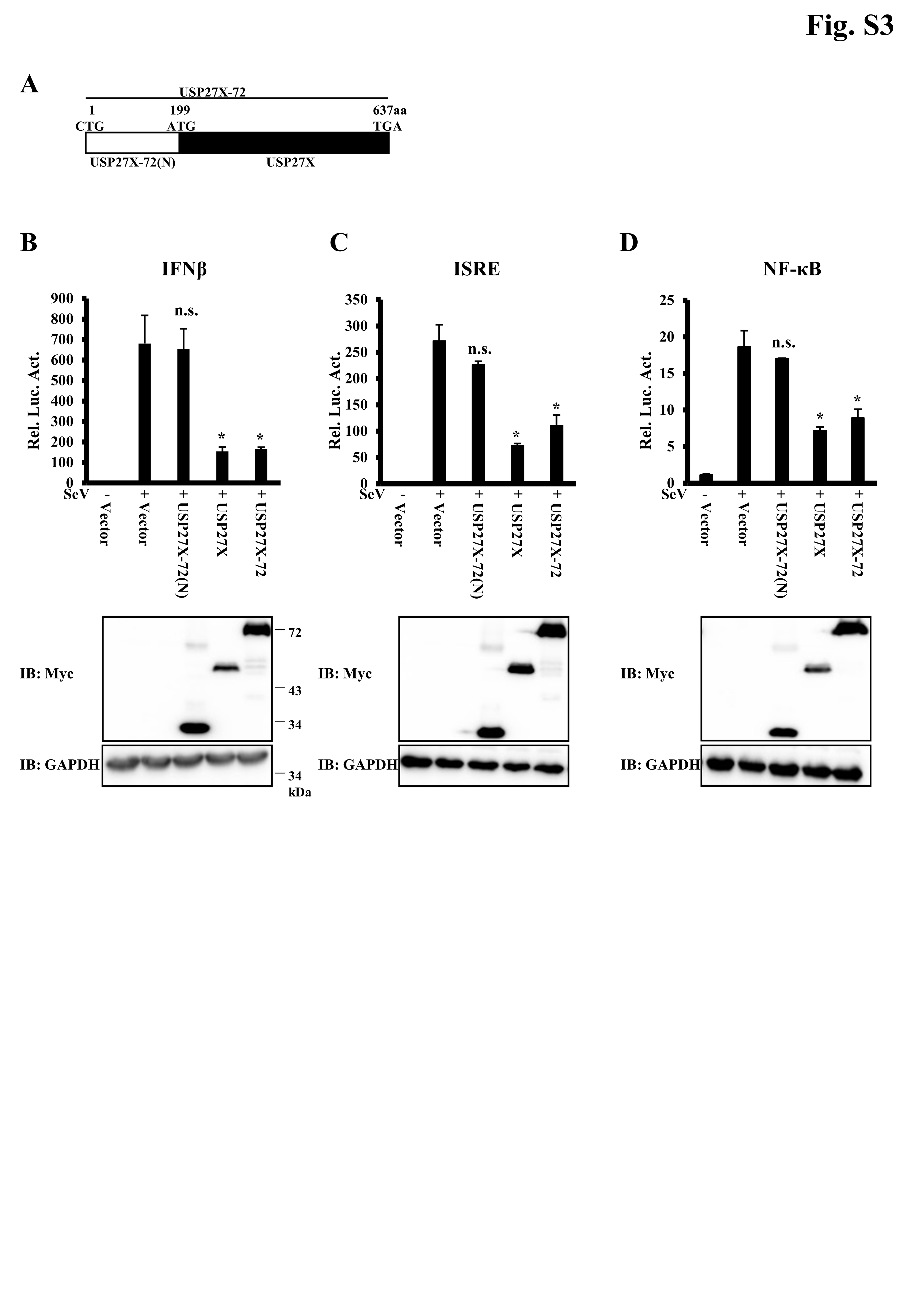

Supplement: S3 Fig — (A) Schematic diagram of USP27X-72. (B–D) HEK293T cells were co-transfected with the indicated expression plasmids along with luciferase reporter constructs driven by promoters of IFNβ (B), ISRE (C) or NF-κB (D). Twenty-four hours after transfection, the cells were infected with SeV for 12 h. The cells were lysed for luciferase assays (upper panel) and immunoblotting assays (lower panels). The data shown in (B–D) are from one representative experiment of at least three independent experiments (mean ± SD of duplicate experiments). The two-tailed Student’s t-test was used to analyze statistical significance. *P < 0.05; n.s. not significant versus control groups. (TIF) [file ppat.1008293.s003.tif]

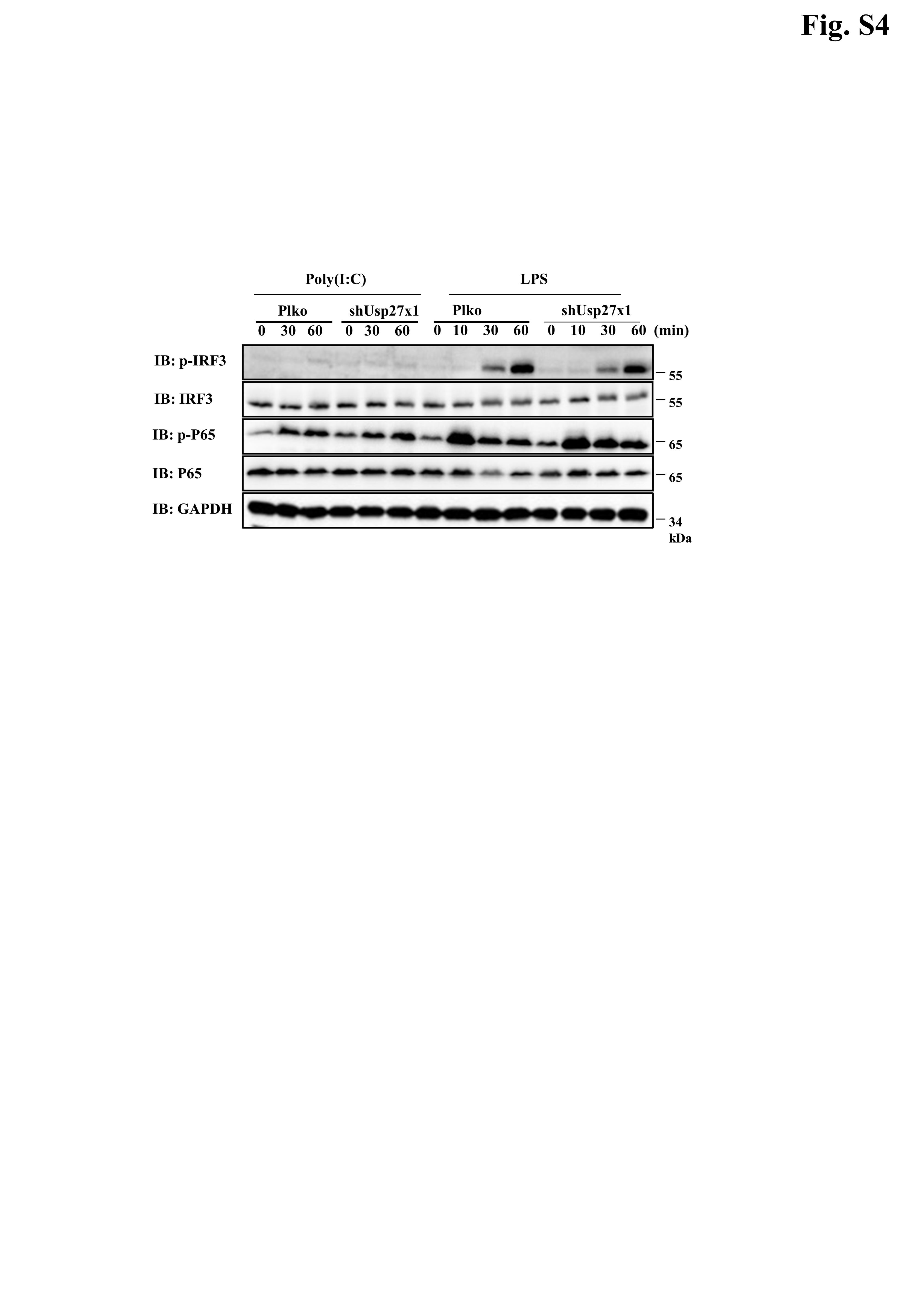

Supplement: S4 Fig — RAW264.7 cells were infected with lentiviral vectors targeting Usp27x (shUsp27x1) or empty vector for 48 h, followed by stimulation with Poly(I:C) or LPS for the indicated times. The cells were lysed for immunoblotting with the indicated antibodies. (TIF) [file ppat.1008293.s004.tif]

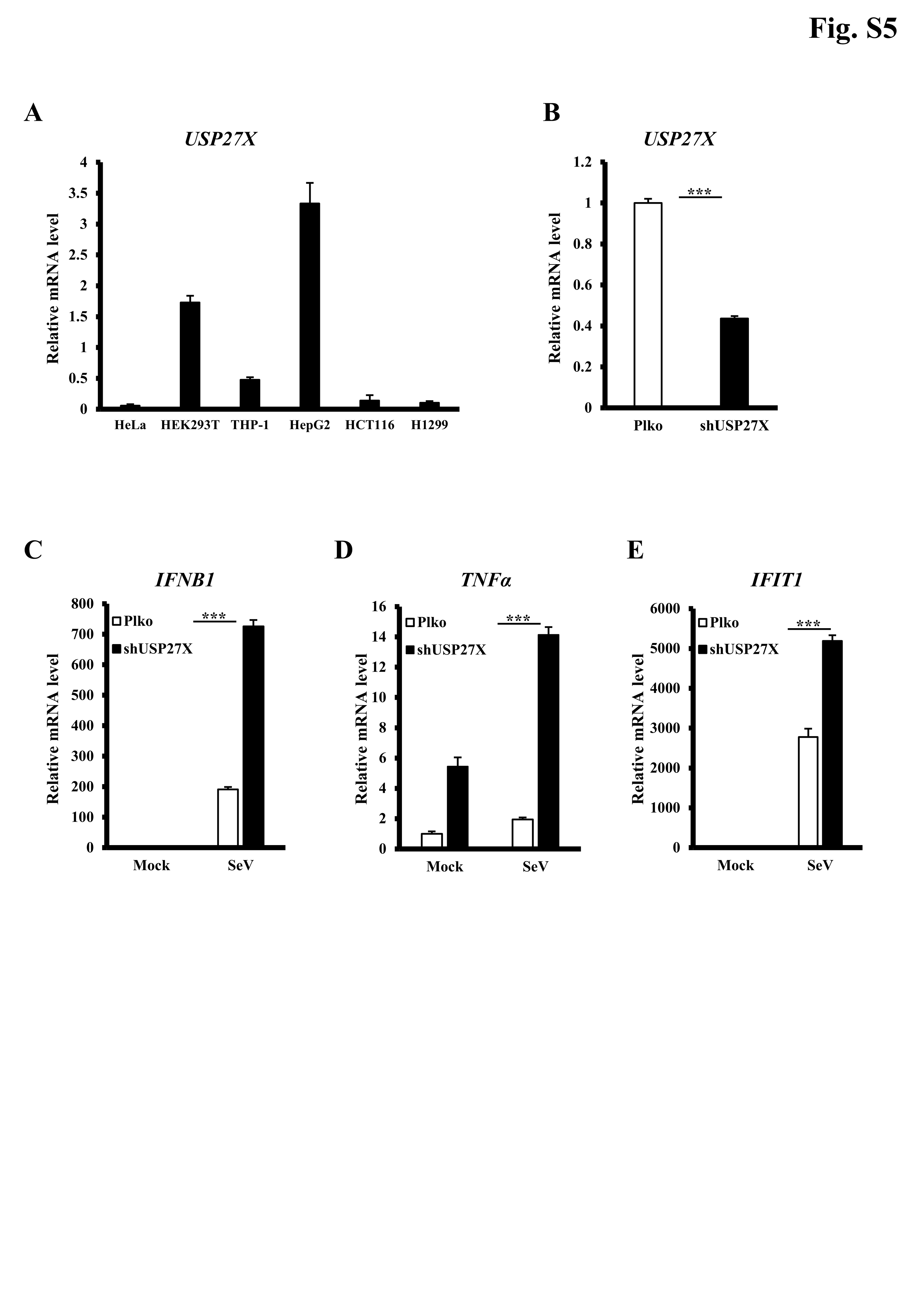

Supplement: S5 Fig — (A) qRT-PCR assays were performed to measure levels of USP27X mRNA in a number of cell lines. (B–E) HepG2 cells were infected with lentiviral vectors targeting USP27X (shUSP27X) or empty vector for 48 h, followed by SeV infection for 12 h. The cells were collected for qRT-PCR assays to measure mRNA levels of USP27X (B), IFNB1 (C), TNFα (D) and IFIT1 (E). The data shown in (A–E) are from one representative experiment of at least three independent experiments (mean ± SD of triplicate experiments). The two-tailed Student’s t-test was used to analyze statistical significance. ***P < 0.001 versus control groups. (TIF) [file ppat.1008293.s005.tif]

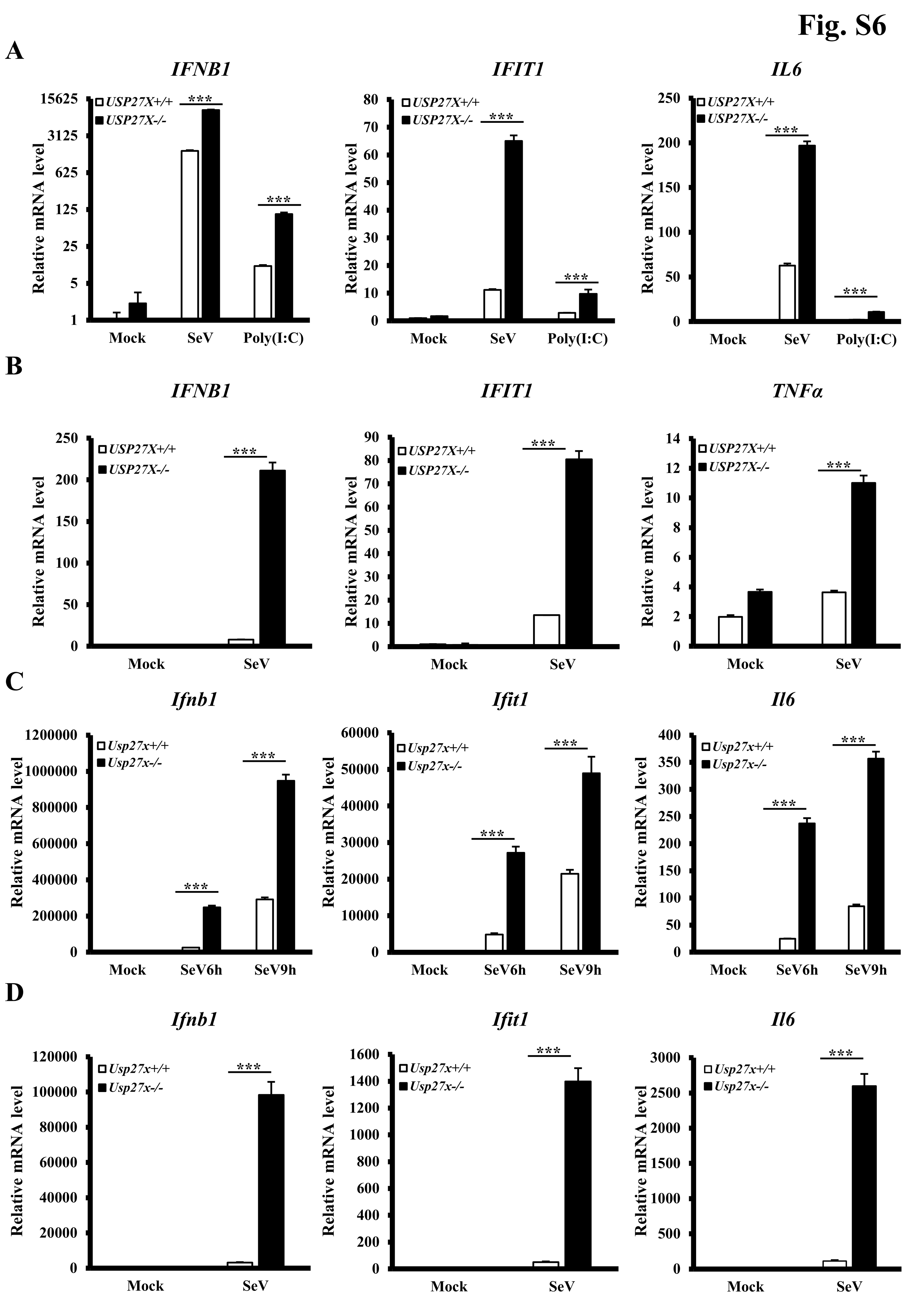

Supplement: S6 Fig — (A–B) HeLa (A) or HepG2 (B) USP27X+/+ and USP27X-/- cells were infected with SeV for 9 h or transfected with Poly(I:C) for 6 h, then lysed for measurement of IFNB1, IFIT1, and IL6 or TNFα mRNA levels by qRT-PCR. (C) L929 Usp27x+/+ and Usp27x-/- cells were infected with SeV for the indicated times, then lysed for measurement of Ifnb1, Ifit1 and Il6 mRNA levels by qRT-PCR. (D) RAW264.7 Usp27x+/+ and Usp27x-/- cells were infected with SeV for 6 h, then lysed for measurement of Ifnb1, Ifit1 and Il6 mRNA levels by qRT-PCR. The data shown in (A–D) are from one representative experiment of at least three independent experiments (mean ± SD of triplicate experiments). The two-tailed Student’s t-test was used to analyze statistical significance. *** P < 0.001 versus control groups. (TIF) [file ppat.1008293.s006.tif]

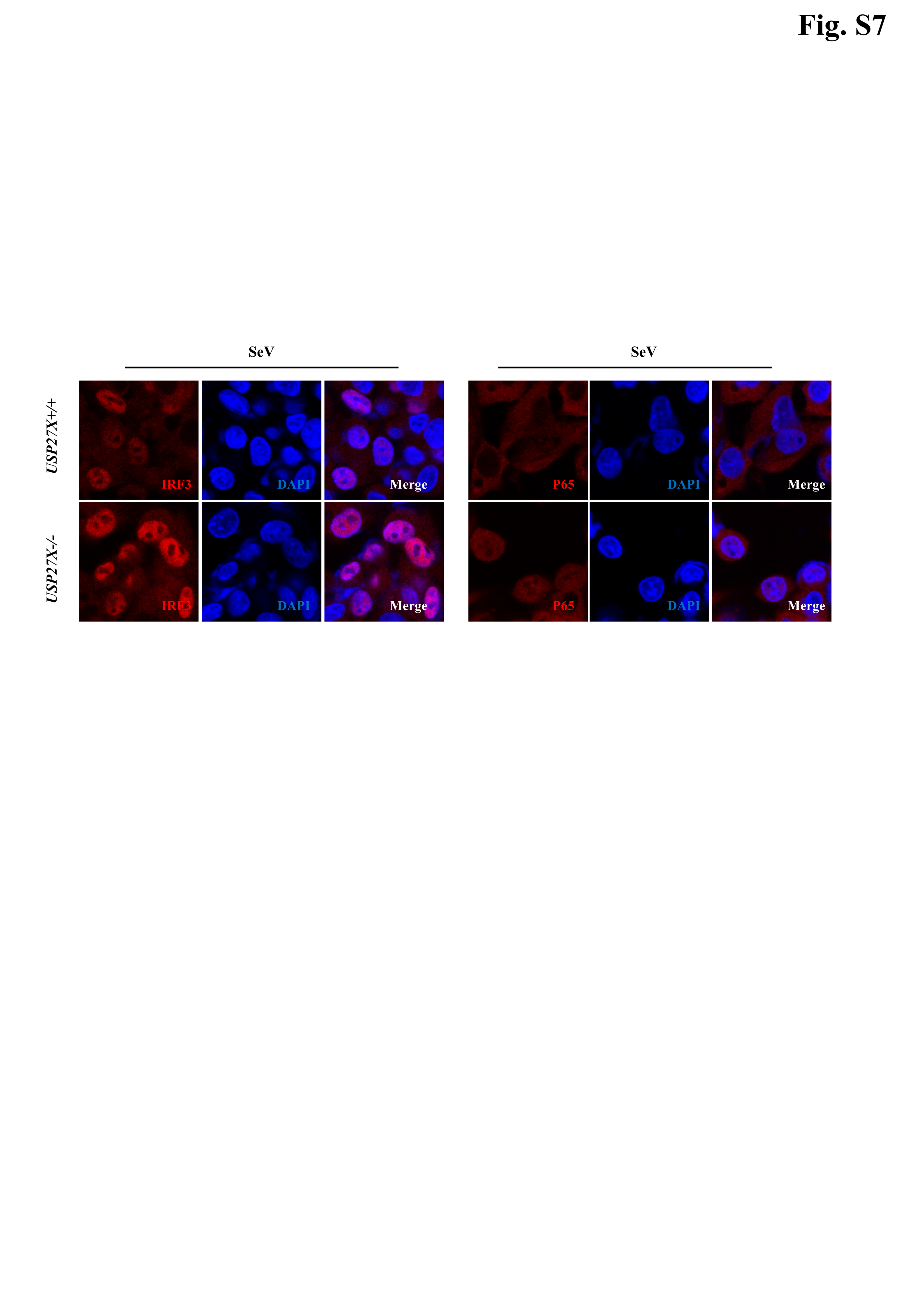

Supplement: S7 Fig — HepG2 USP27X+/+ and USP27X-/- cells were mock-infected or infected with SeV (100HA) for 9 h. The cells were fixed, stained with the anti-IRF3 (red) (left panels) or anti-P65 (red) (right panels) antibodies, and observed by confocal microscopy. (TIF) [file ppat.1008293.s007.tif]

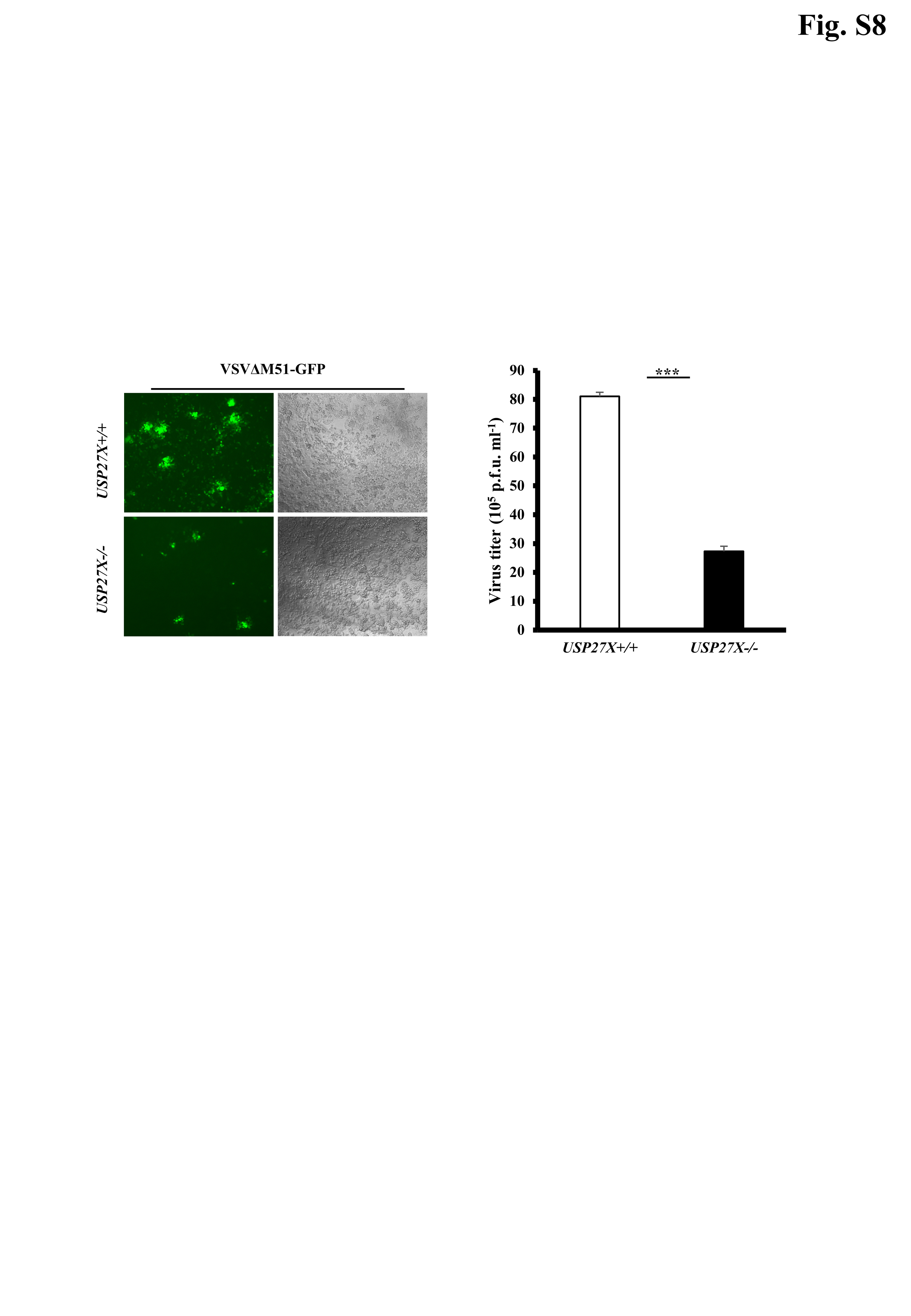

Supplement: S8 Fig — HepG2 USP27X +/+ and USP27X-/- cells were infected with VSVΔM51-GFP at an MOI of 0.01 for 12 h. Culture supernatants were collected to measure viral titers by plaque assay. The data shown in the right panel are from one representative experiment of at least three independent experiments (mean ± SD duplicate experiments). The two-tailed Student’s t-test was used to analyze statistical significance. *** P < 0.001 versus control groups. (TIF) [file ppat.1008293.s008.tif]

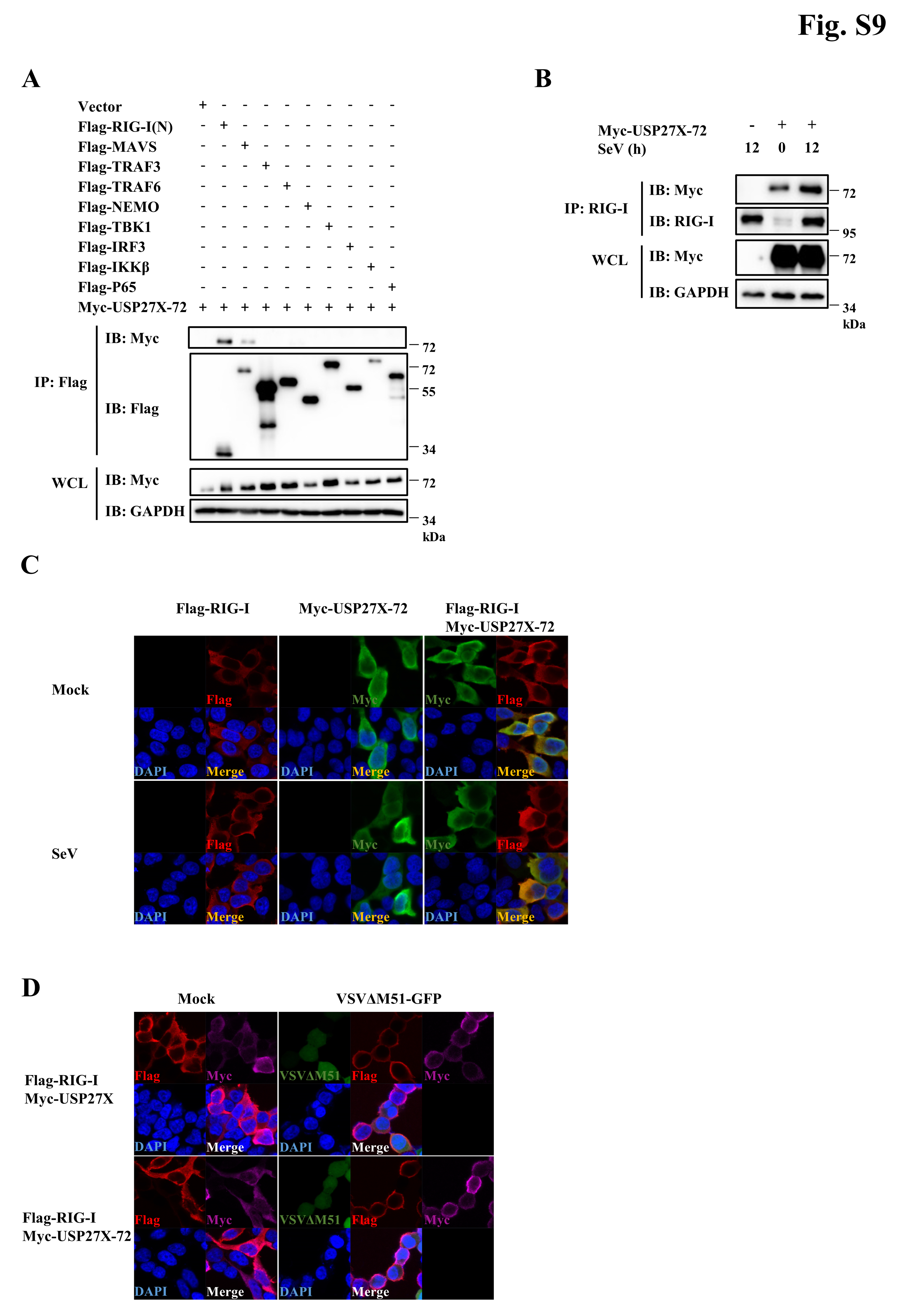

Supplement: S9 Fig — (A) HEK293T cells were transfected with the indicated expression plasmids. Twenty-four hours after transfection, the cells were lysed for Co-IP with anti-Flag agarose beads, followed by immunoblotting. The expression levels of transfected proteins in whole cell lysates (WCL) are shown in the bottom panels. (B) HEK293T cells were transfected with Myc-USP27X-72 expression vector or empty vector. Twenty-four hours after transfection, the cells were mock-infected or infected with SeV for 12 h. Cell lysates were immunoprecipitated with anti-RIG-I antibody, followed by immunoblotting. (C) HEK293T cells were transfected with the indicated expression plasmids. Twenty-four hours after transfection, cells were mock-infected or infected with SeV (50HA) for 9 h. The cells were fixed, stained with the anti-Flag (red) and anti-Myc (green) antibodies, and observed by confocal microscopy. (D) HEK293T cells were transfected with the indicated expression plasmids. Twenty-four hours after transfection, cells were mock-infected or infected with VSVΔM51-GFP (1 MOI) for 9 h. The cells were fixed, stained with the anti-Flag (red) and anti-Myc (pink) antibodies, and observed by confocal microscopy. (TIF) [file ppat.1008293.s009.tif]

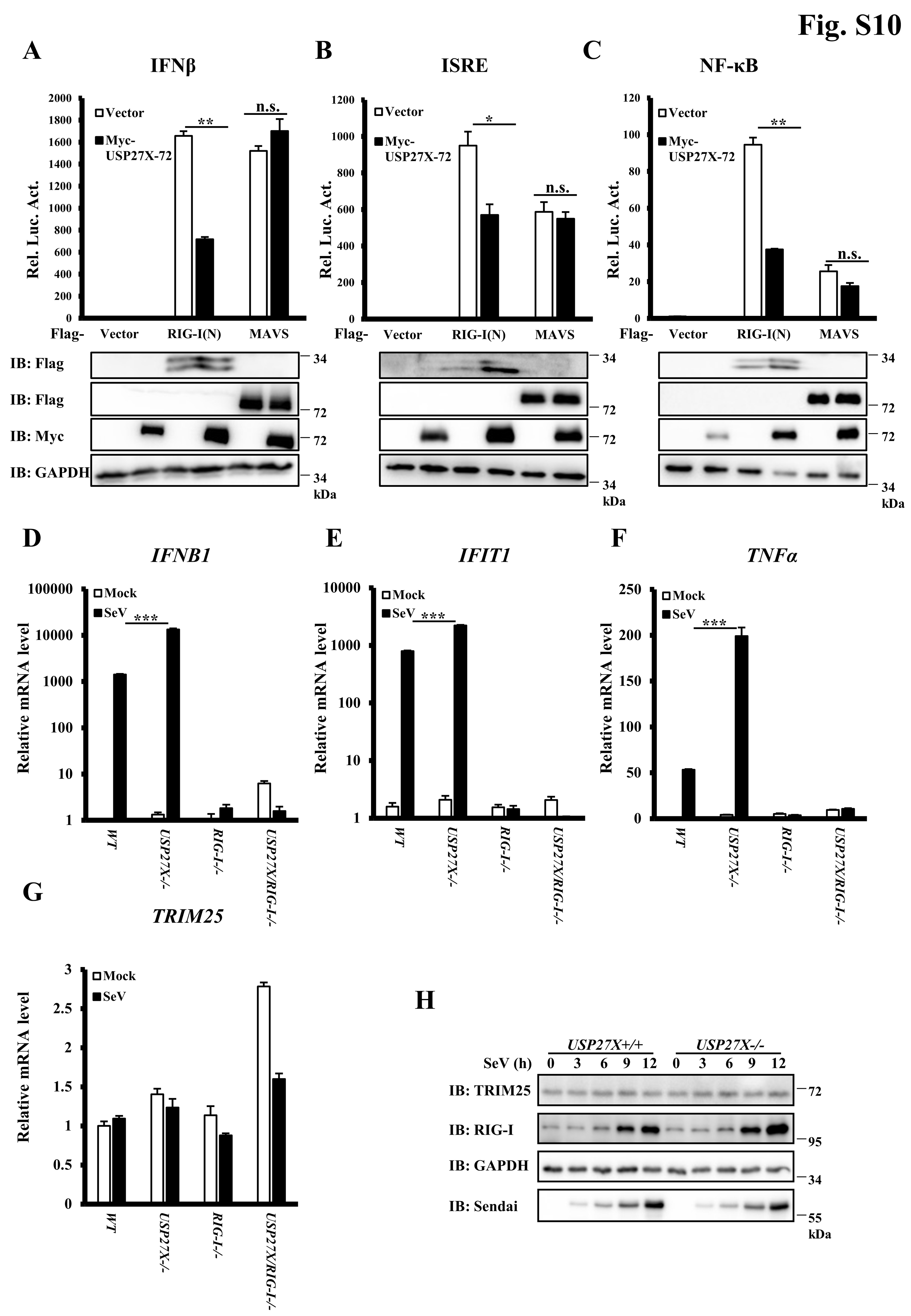

Supplement: S10 Fig — (A–C) HEK293T cells were co-transfected with the indicated expression plasmids along with luciferase reporter constructs driven by promoters of IFNβ (A), ISRE (B) or NF-κB (C) as well as Renilla as an internal control. Twenty-four hours after transfection, the cells were lysed for luciferase assays (upper panel) and immunoblotting assays (lower panels). (D–G) HEK293T WT, USP27X-/-, RIG-I-/- and USP27X/RIG-I-/- cells were infected with SeV for 9 h, then lysed for measurement of IFNB1 (D), IFIT1 (E), TNFα (F) and TRIM25 (G) mRNA levels by qRT-PCR. (H) HeLa USP27X+/+ and USP27X-/- cells were infected with SeV for the indicated times, then lysed for immunoblotting with the indicated antibodies. The data shown in (A–G) are from one representative experiment of at least three independent experiments [mean ± SD of duplicate experiments in (A–C) or triplicate experiments in (D–G)]. The two-tailed Student’s t-test was used to analyze statistical significance. * P < 0.05; **P < 0.01; ***P < 0.001; n.s. not significant versus control groups. (TIF) [file ppat.1008293.s010.tif]

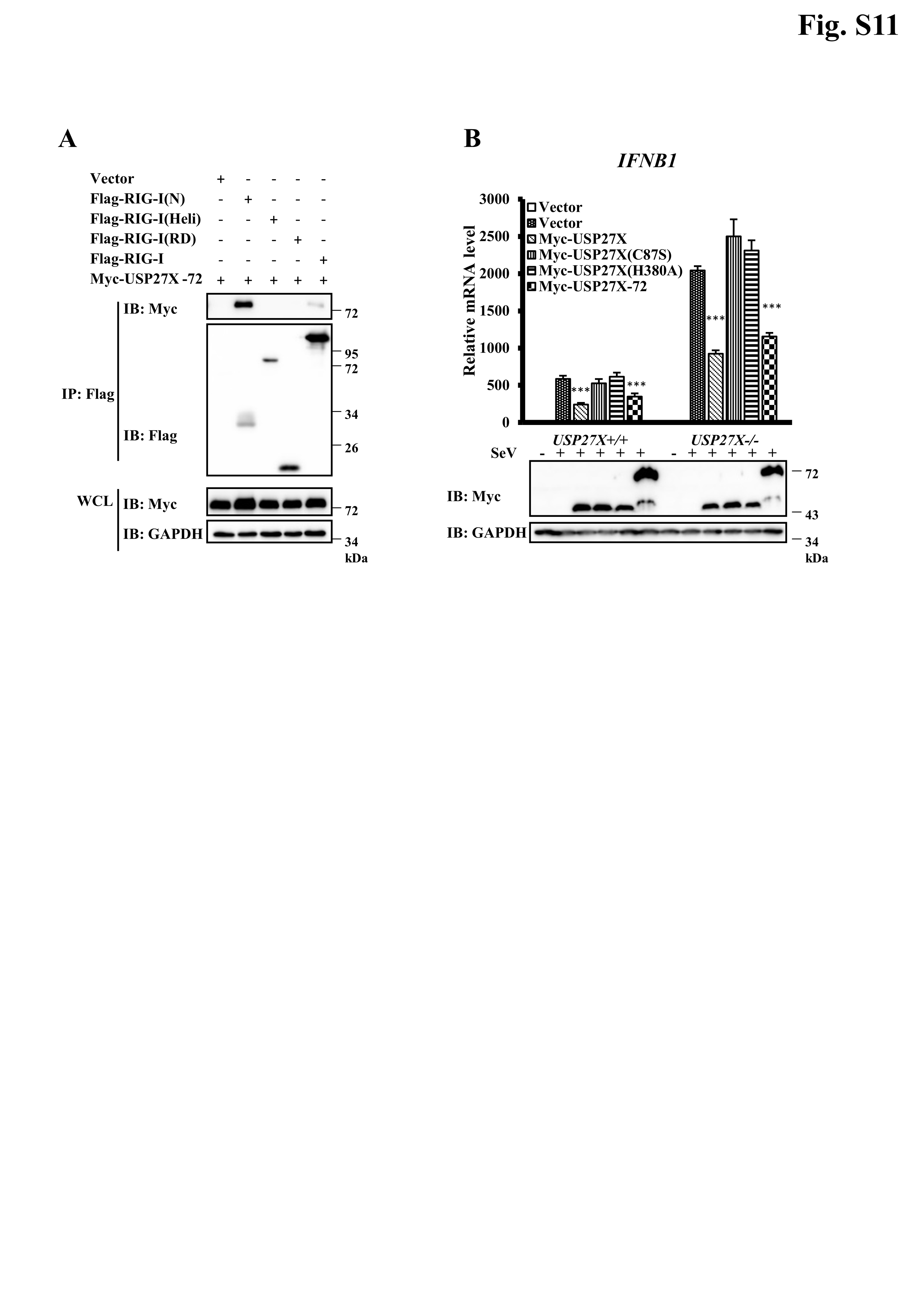

Supplement: S11 Fig — (A) HEK293T cells were transfected with the indicated expression plasmids. Cell lysates were immunoprecipitated with anti-Flag beads, followed by immunoblotting. (B) HEK293T USP27X+/+ and USP27X-/- cells were transfected with indicated expression plasmids. Twenty-four hours after transfection, the cells were infected with SeV for 9 h, followed by measurement of IFNB1 mRNA levels by qRT-PCR. The data shown in (B) are from one representative experiment of at least three independent experiments (mean ± SD of triplicate experiments). The two-tailed Student’s t-test was used to analyze statistical significance. *** P < 0.001 versus control groups. (TIF) [file ppat.1008293.s011.tif]

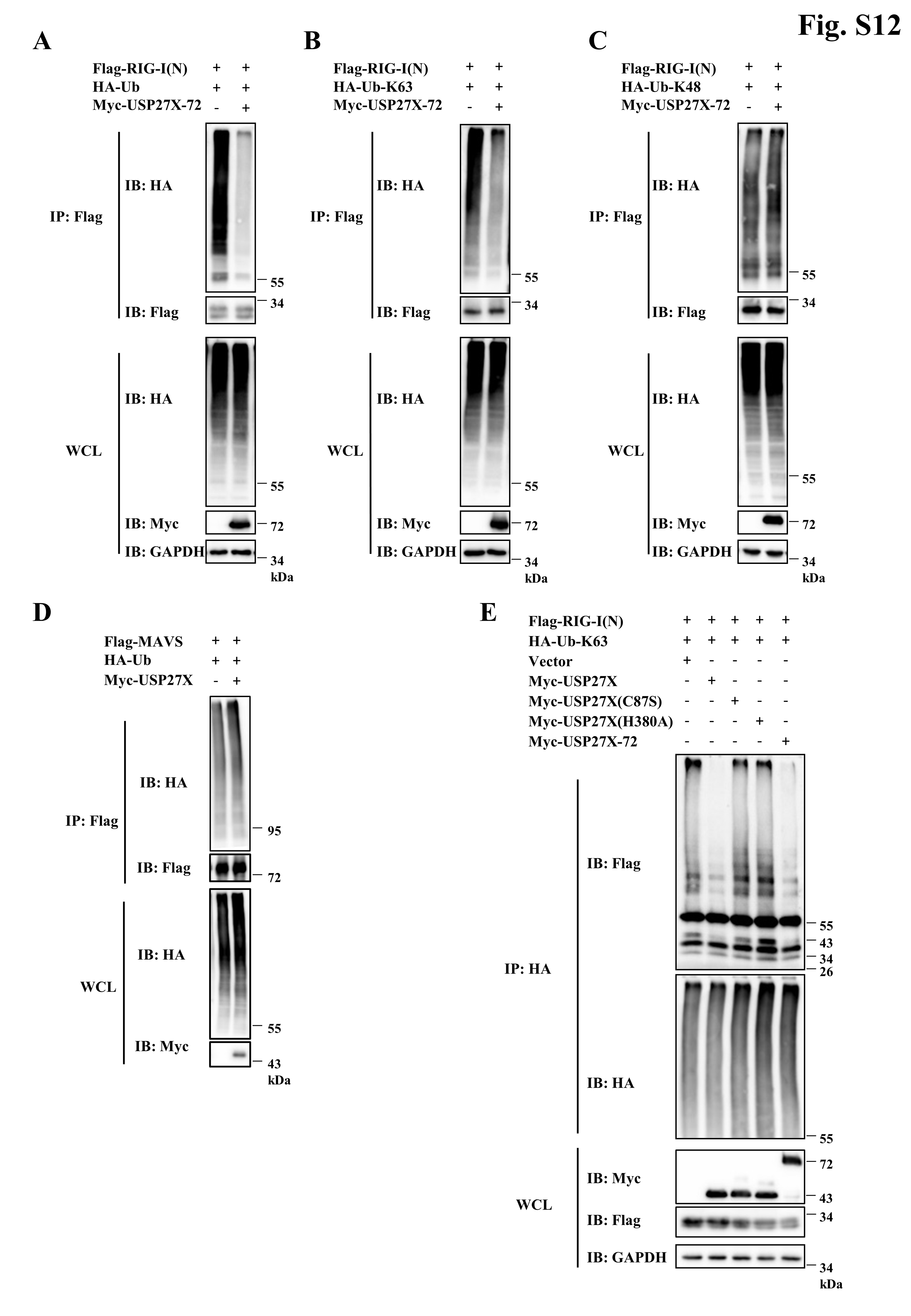

Supplement: S12 Fig — (A–E) HEK293T cells were transfected with the indicated plasmids. Cell lysates were immunoprecipitated with anti-Flag or anti-HA beads, followed by immunoblotting with the indicated antibodies. (TIF) [file ppat.1008293.s012.tif]

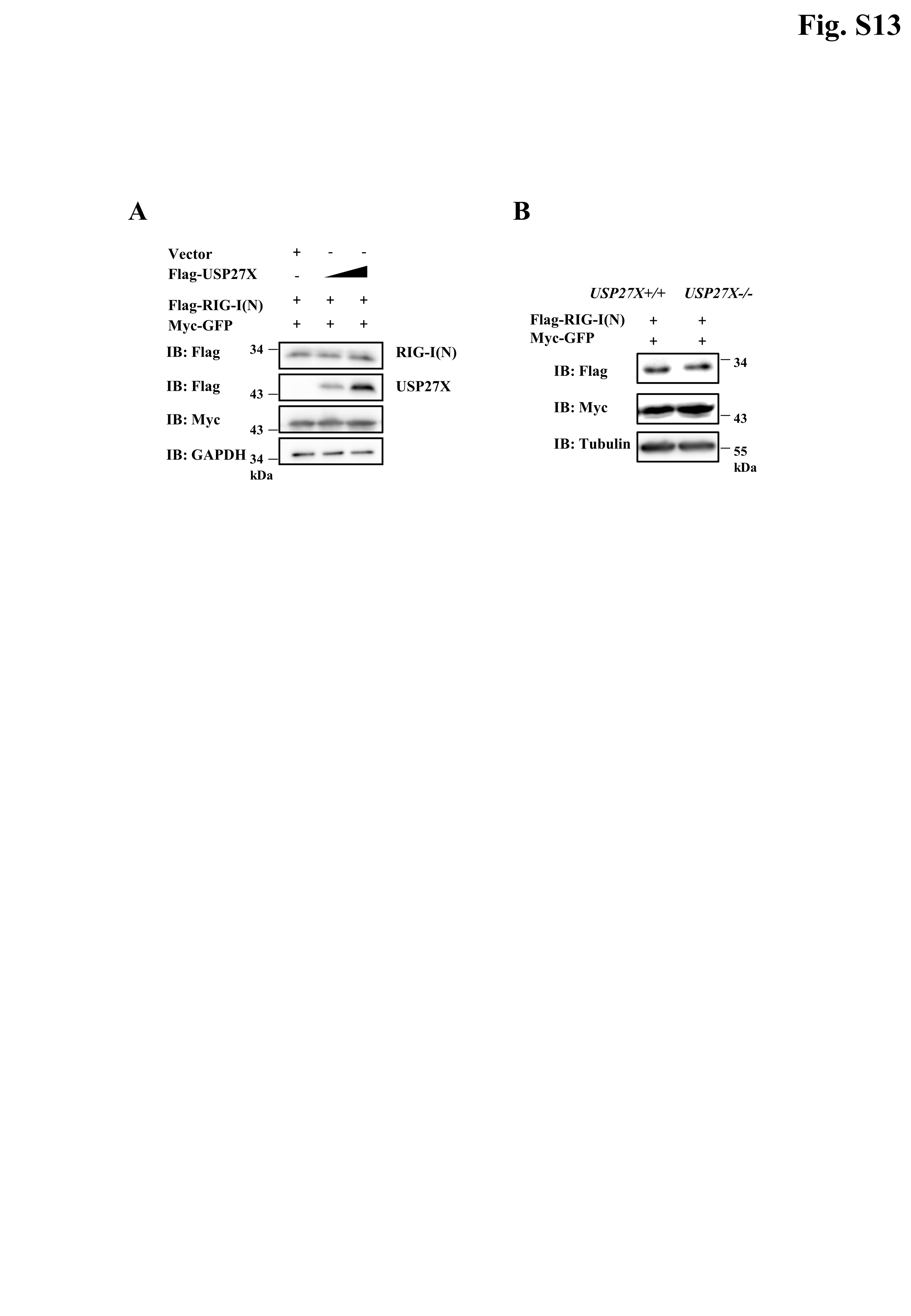

Supplement: S13 Fig — (A) HEK293T cells were transfected with the indicated plasmids. Twenty-four hours after transfection, the cells were lysed for immunoblotting with the indicated antibodies. (B) HEK293T USP27X+/+ and USP27X-/- cells were co-transfected with Myc-GFP and Flag-RIG-I(N) plasmids. Twenty-four hours after transfection, cell lysates were for immunoblotting analysis with the indicated antibodies. (TIF) [file ppat.1008293.s013.tif]

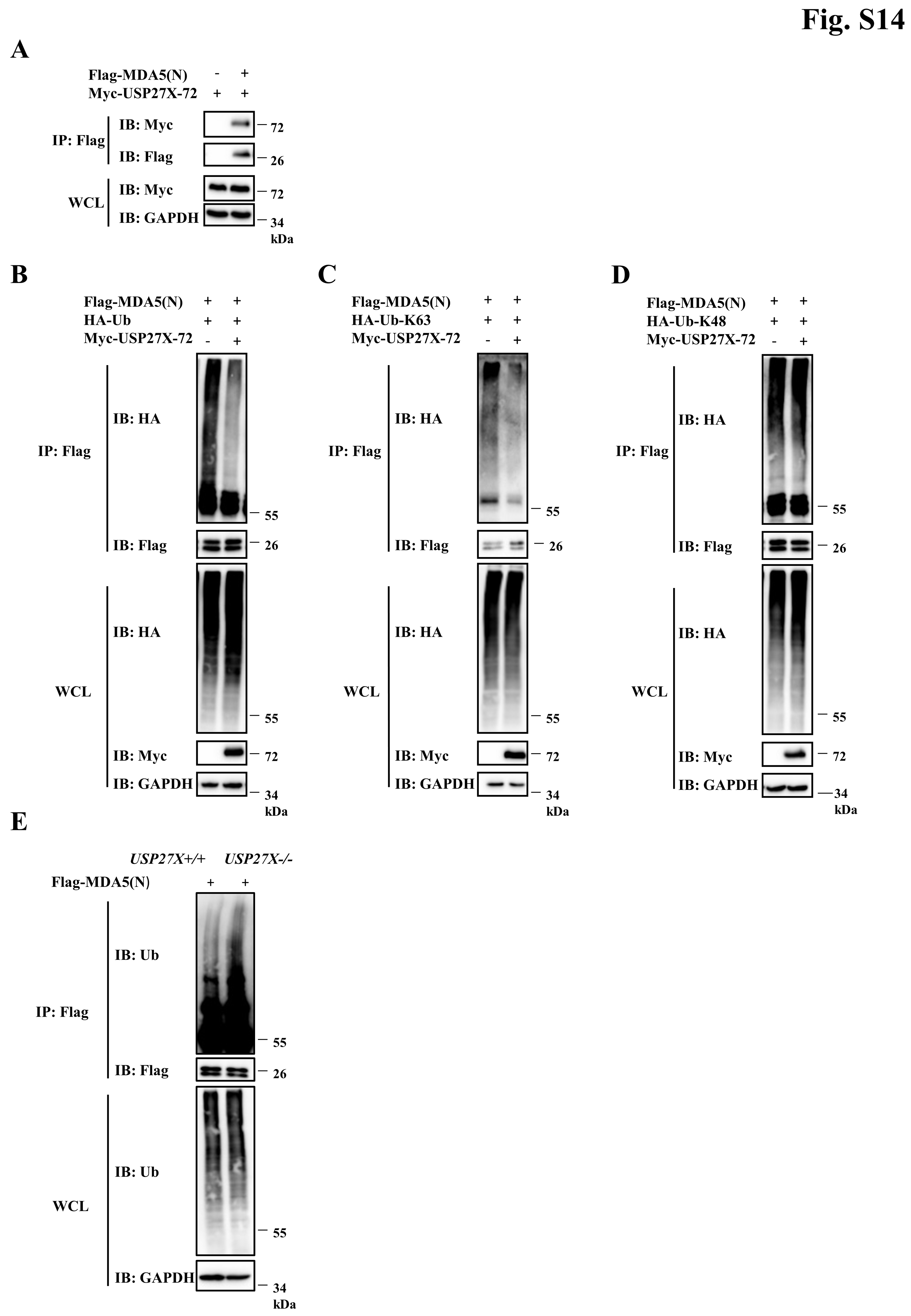

Supplement: S14 Fig — (A) HEK293T cells were transfected with the indicated expression plasmids. Cell lysates were immunoprecipitated with anti-Flag beads, followed by immunoblotting. (B–D) HEK293T cells were transfected with USP27X-72 and MDA5(N) together with HA-tagged wild-type Ub (HA-Ub) (B), HA-Ub-K63 (C), or HA-Ub-K48 (D) plasmids. Twenty-four hours after transfection, cell lysates were immunoprecipitated with anti-Flag beads, followed by immunoblotting analysis with the indicated antibodies. The expression levels of transfected proteins in whole cell lysates (WCL) are shown in the bottom panels. (E) HepG2 USP27X+/+ and USP27X-/- cells were transfected with Flag-MDA5(N) plasmids. Twenty-four hours after transfection, cell lysates were immunoprecipitated with anti-Flag beads, followed by immunoblotting analysis with the indicated antibodies. The expression levels of transfected proteins in whole cell lysates (WCL) are shown in the bottom panels. (TIF) [file ppat.1008293.s014.tif]

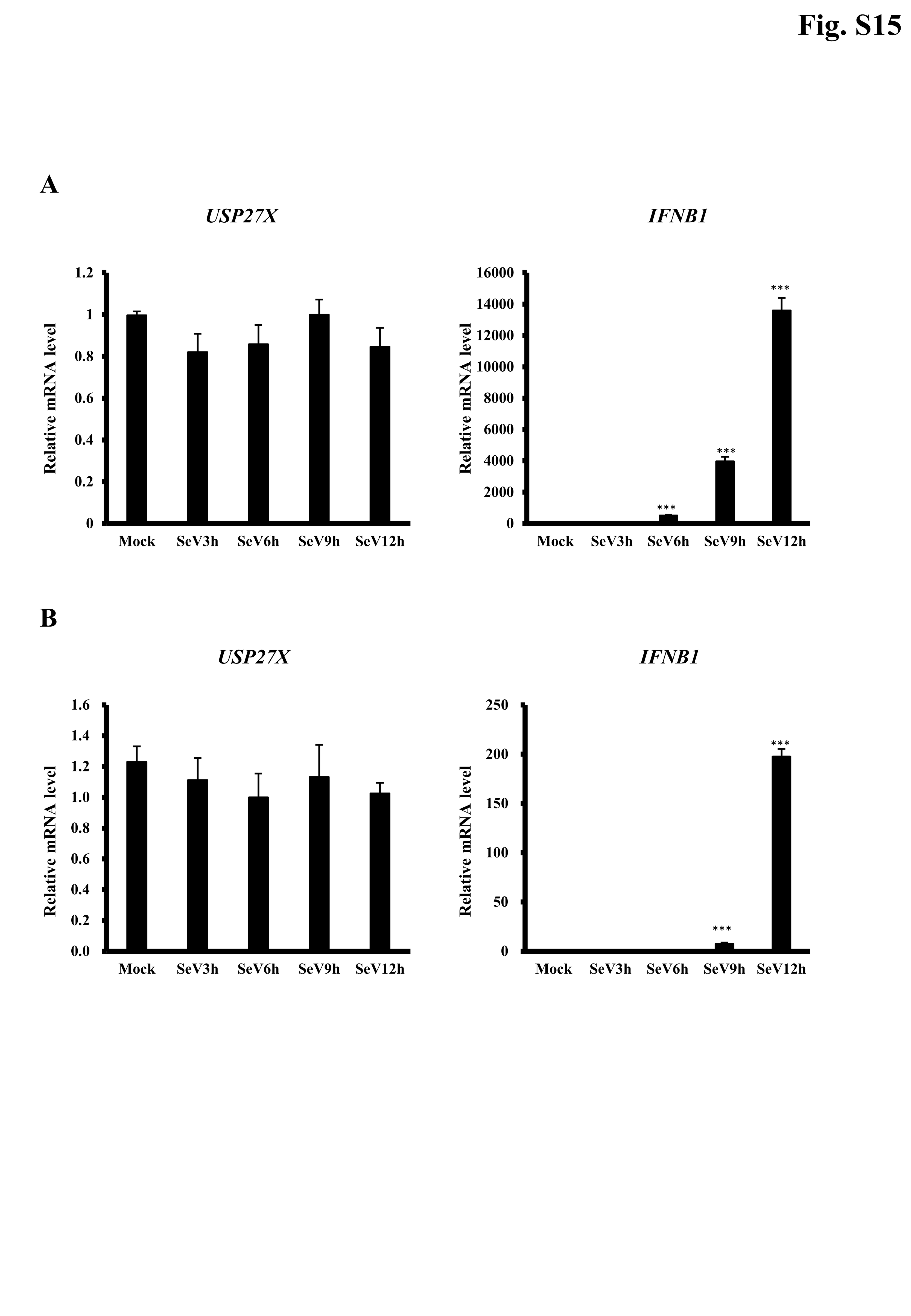

Supplement: S15 Fig — HEK293T (A) or HepG2 (B) were infected with SeV for the indicated times, then lysed for measurement of USP27X and IFNB1 mRNA levels by qRT-PCR. The data shown in (A–B) are from one representative experiment of at least three independent experiments (mean ± SD of triplicate experiments). The two-tailed Student’s t-test was used to analyze statistical significance. ***P < 0.001, versus control groups. (TIF) [file ppat.1008293.s015.tif]

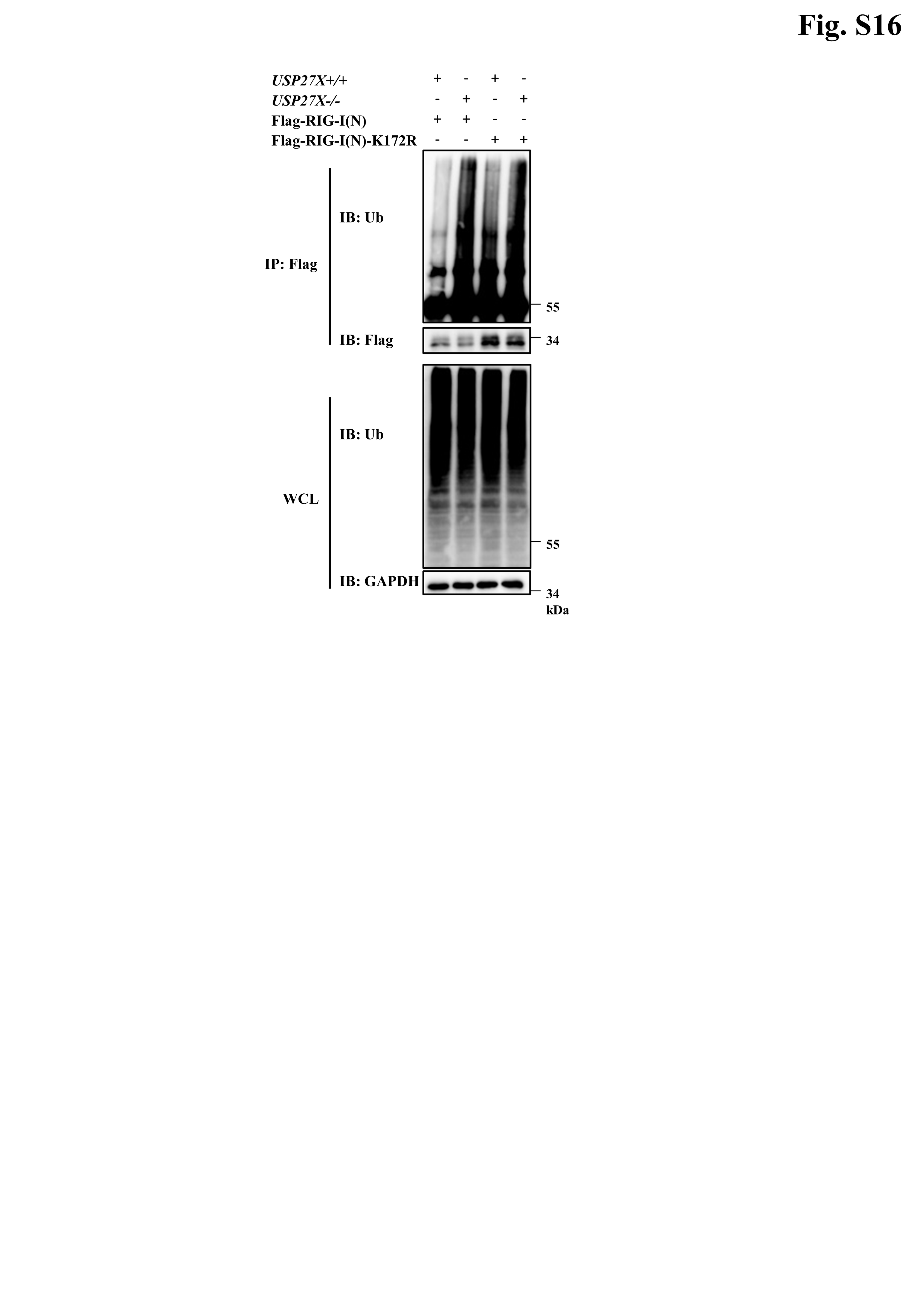

Supplement: S16 Fig — HepG2 USP27X+/+ and USP27X-/- cells were transfected with Flag-RIG-I(N) or Flag-RIG-I(N)-K172R plasmids. Twenty-four hours after transfection, cell lysates were immunoprecipitated with anti-Flag beads, followed by immunoblotting analysis with the indicated antibodies. The expression levels of transfected proteins in whole cell lysates (WCL) are shown in the bottom panels. (TIF) [file ppat.1008293.s016.tif]

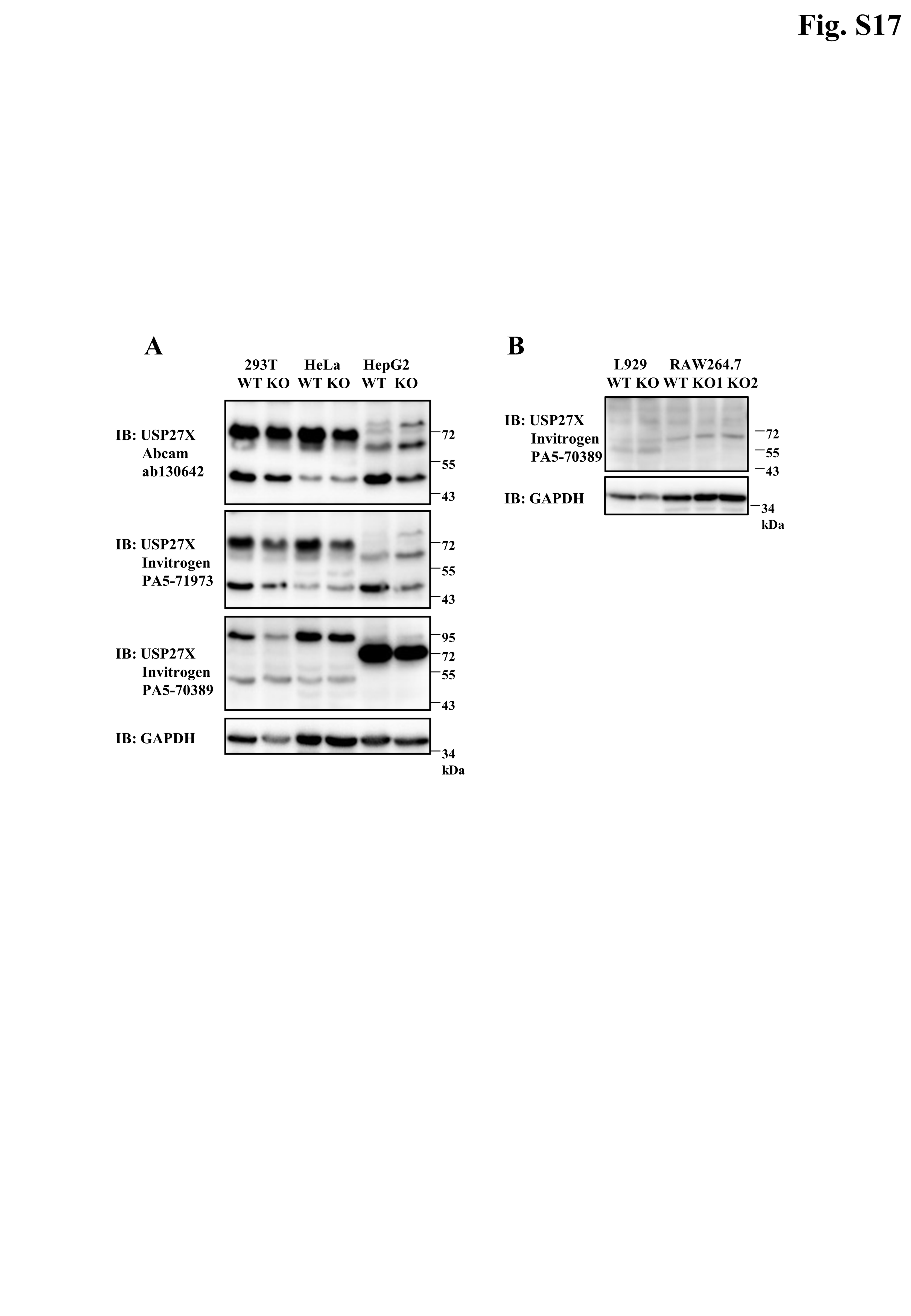

Supplement: S17 Fig — (A) USP27X+/+ and USP27X-/- cells including HEK293T, HeLa and HepG2 cells were lysed for immunoblotting with the indicated antibodies. (B) Usp27x+/+ and Usp27x-/- cells including L929 and RAW264.7 cells were lysed for immunoblotting with the indicated antibodies. (TIF) [file ppat.1008293.s017.tif]

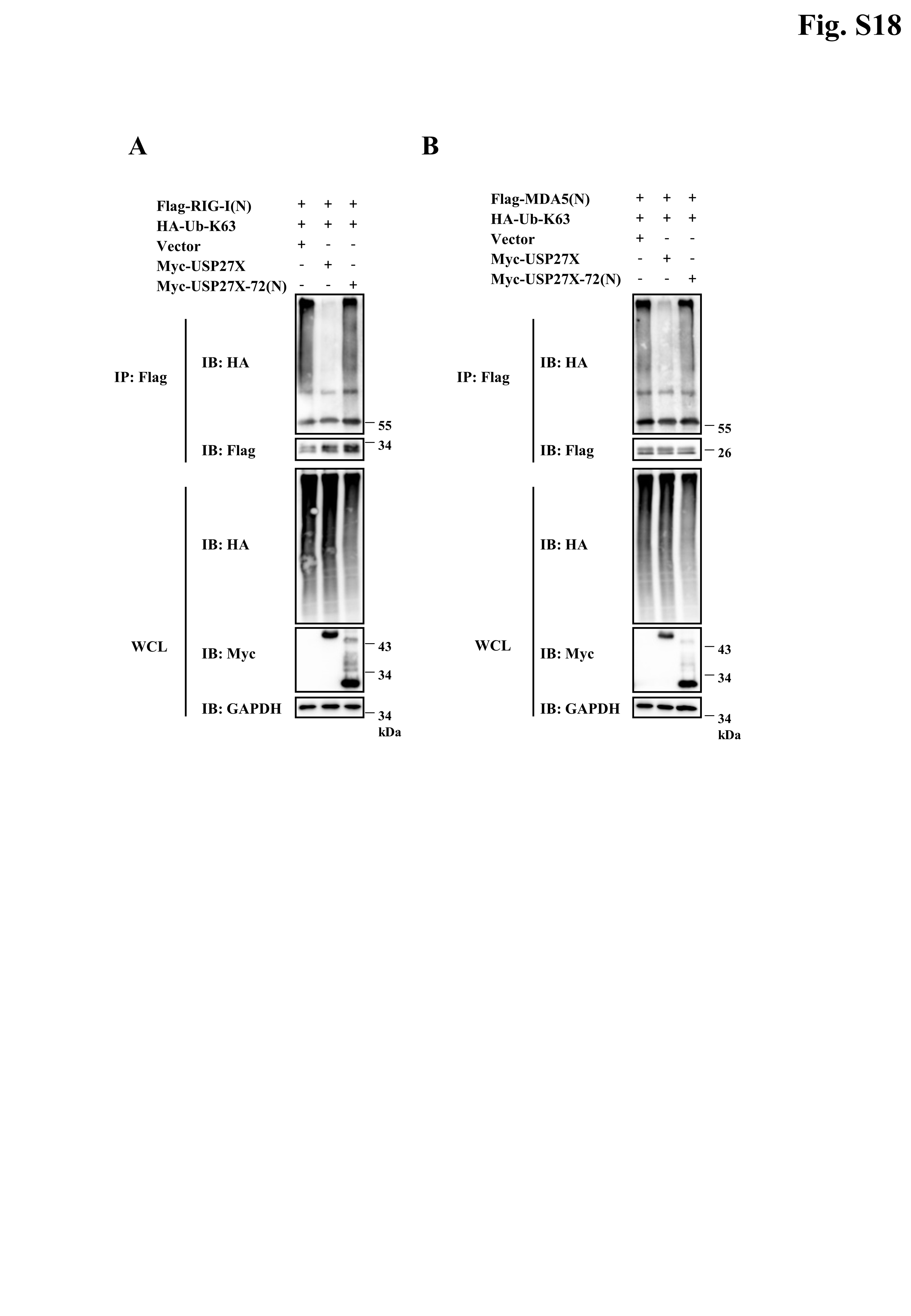

Supplement: S18 Fig — (A–B) HEK293T cells were transfected with USP27X, USP27X-72(N) and RIG-I(N) (A) or MDA5(N) (B) together with HA-tagged Ub-K63 plasmids. Twenty-four hours after transfection, cell lysates were immunoprecipitated with anti-Flag beads, followed by immunoblotting analysis with the indicated antibodies. The expression levels of transfected proteins in whole cell lysates (WCL) are shown in the bottom panels. (TIF) [file ppat.1008293.s018.tif]

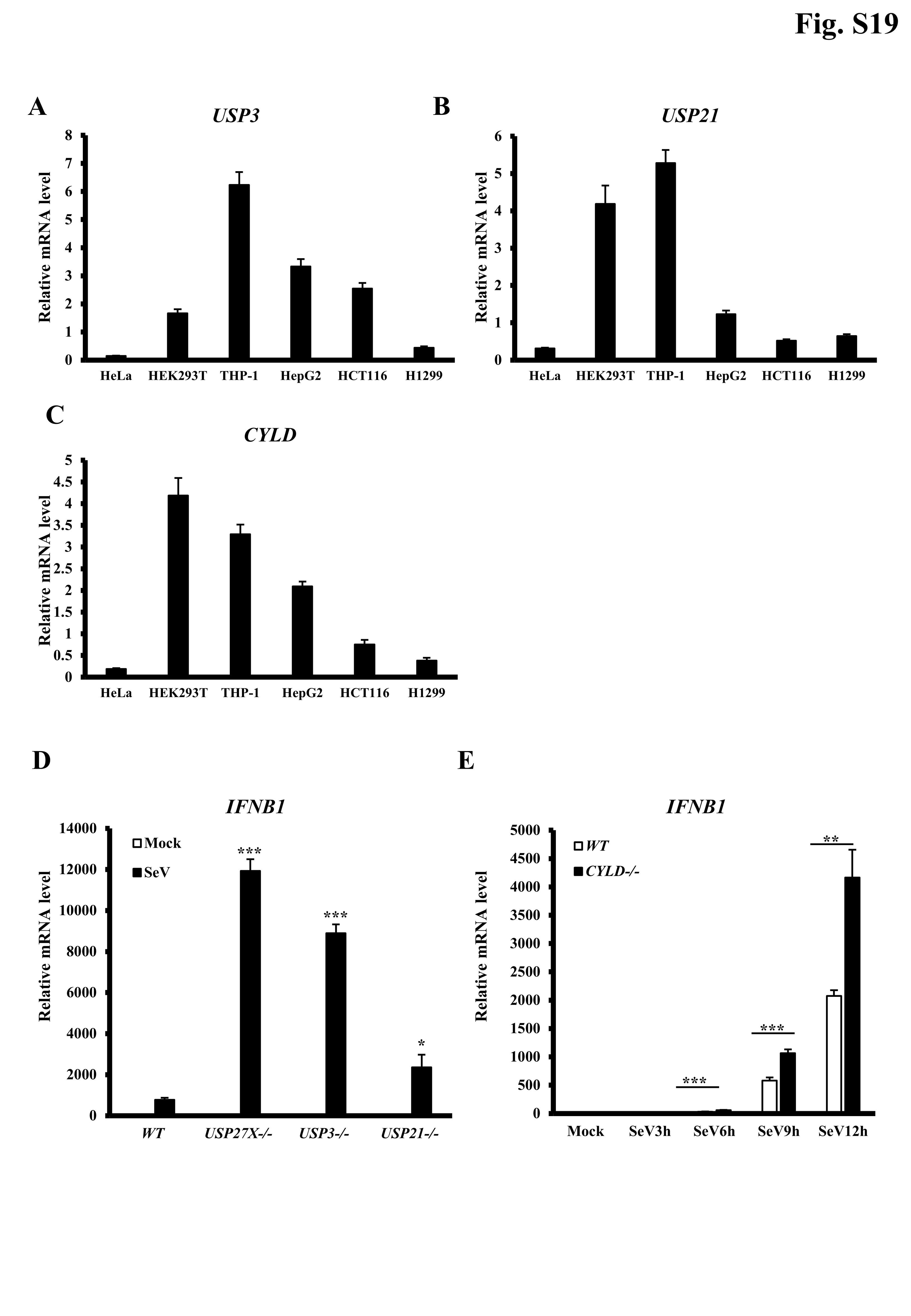

Supplement: S19 Fig — (A–C) qRT-PCR assays were performed to measure levels of USP3 (A), USP21 (B) and CYLD (C) mRNA in a number of cell lines. (D) HEK293T WT, USP27X-/-, USP3-/- and USP21-/- cells were infected with SeV for 9 h, then lysed for measurement of IFNB1 mRNA levels by qRT-PCR. (E) HEK293T WT and CYLD-/- cells were infected with SeV for the indicated times, then lysed for measurement of IFNB1 mRNA levels by qRT-PCR. The data shown in (A–E) are from one representative experiment of at least three independent experiments (mean ± SD of triplicate experiments). The two-tailed Student’s t-test was used to analyze statistical significance. * P < 0.05; **P < 0.01; ***P < 0.001, versus control groups. (TIF) [file ppat.1008293.s019.tif]

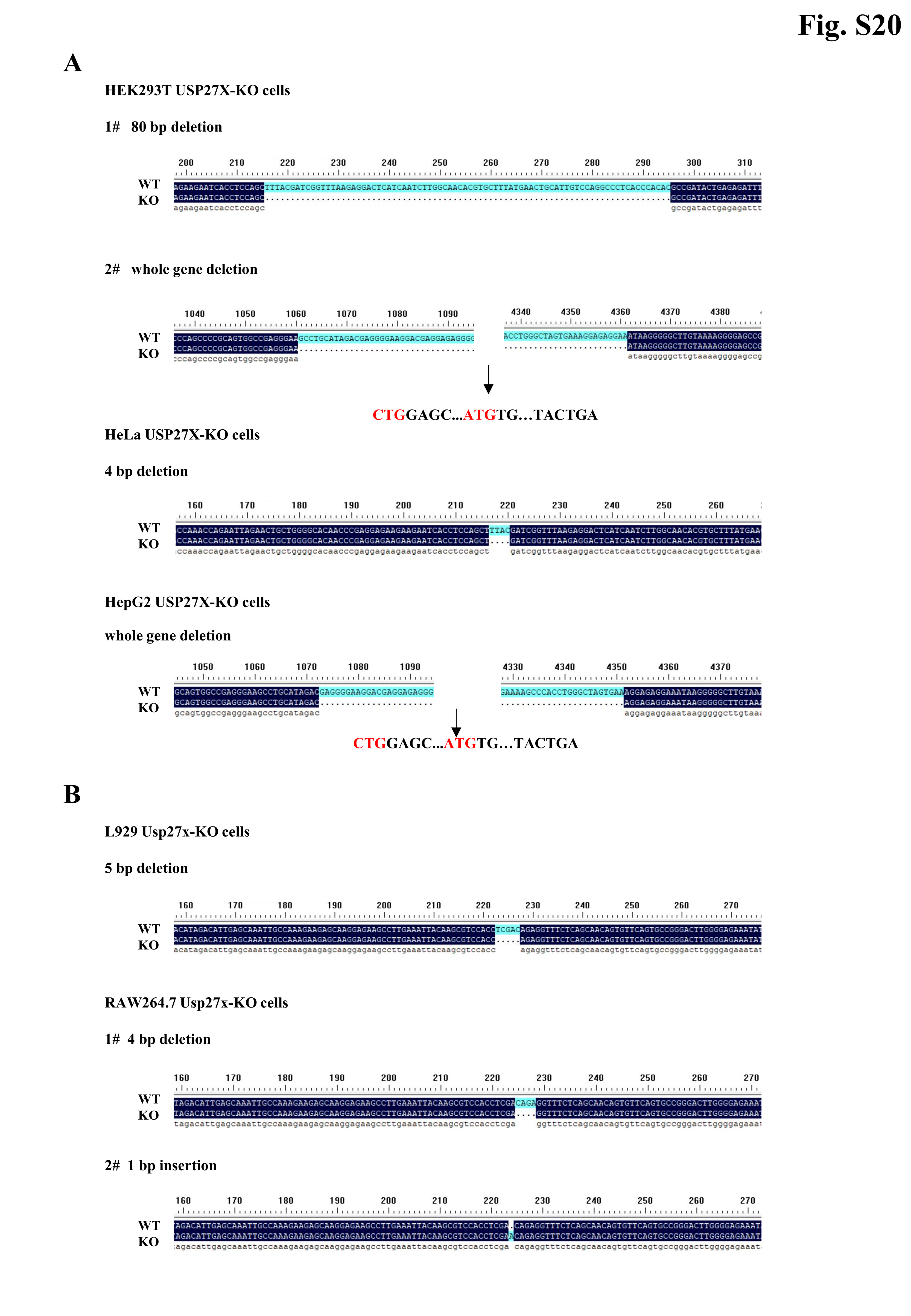

Supplement: S20 Fig — USP27X-KO cells including HEK293T, HeLa, HepG2, L929 and RAW264.7 cells were generated by CRISPR/Cas9 gene editing system, and USP27X-KO cells were verified by DNA sequencing. (TIF) [file ppat.1008293.s020.tif]
